# Association of meat consumption with the risk of gastrointestinal cancers: a systematic review and meta-analysis

**DOI:** 10.1186/s12885-023-11218-1

**Published:** 2023-08-23

**Authors:** Yan Di, Lei Ding, Luying Gao, Hongyan Huang

**Affiliations:** 1 Department of Medical Oncology, Beijing Shijitan Hospital, Capital Medical University, Beijing, China; 2 Department of Oncology Surgery/ Beijing Shijitan Hospital, Capital Medical University, Beijing, China; 3 Department of Ultrasond/Peking Union Medical College Hospital, Chinese Academy of Medical Sciences and Peking Union Medical College, Beijing, China

## Abstract

**Background:**

The association between gastrointestinal cancer and types of meat consumption, including red meat, processed meat, or a combination of both, remains disputable. Therefore, we performed a systematic review and meta-analysis of prospective cohort studies to estimate the association between meat consumption and gastrointestinal cancer risk.

**Methods:**

PubMed, EmBase, and the Cochrane library databases were searched systematically for eligible studies that investigated the relation between meat consumption and the risk of developing gastrointestinal cancers, including esophageal cancer (EC), gastric cancer (GC), colorectal cancer (CRC), colon cancer (CC), rectal cancer (RC), pancreatic cancer (PC), and hepatocellular carcinoma (HCC) throughout February, 2023. The pooled relative risk (RR) with 95% confidence interval (CI) was assigned as an effect estimate and calculated using a random-effects model with inverse variance weighting.

**Results:**

Forty cohorts comprising 3,780,590 individuals were selected for the final quantitative analysis. The summary results indicated that a higher red meat consumption was associated with an increased risk of CRC (RR: 1.09; 95% CI: 1.02–1.16; *P* = 0.007) and CC (RR: 1.13; 95% CI: 1.03–1.25; *P* = 0.011). Moreover, a higher processed meat consumption was associated with an increased risk of CRC (RR: 1.19; 95% CI: 1.13–1.26; *P* < 0.001), CC (RR: 1.24; 95% CI: 1.13–1.26; *P* < 0.001), and RC (RR: 1.24; 95% CI: 1.08–1.42; *P* = 0.002). Furthermore, a higher total consumption of red and processed meat was associated with an increased risk of CRC (RR: 1.13; 95% CI: 1.06–1.20; *P* < 0.001), CC (RR: 1.17; 95% CI: 1.04–1.33; *P* = 0.012), and RC (RR: 1.20; 95% CI: 1.04–1.39; *P* = 0.016). Finally, the strength of higher consumption of total red and processed meat with the risk of GC, and higher consumption of red meat with the risk of RC in subgroup of high adjusted level was lower than subgroup of moderate adjusted level, while the strength of higher consumption of processed meat with the risk of RC and HCC in subgroup of follow-up ≥ 10.0 years was higher than subgroup of follow-up < 10.0 years.

**Conclusions:**

This study found that meat consumption was associated with an increased risk of CRC, CC, and RC, and dietary intervention could be considered an effective strategy in preventing CRC.

**Supplementary Information:**

The online version contains supplementary material available at 10.1186/s12885-023-11218-1.

## Background

Gastrointestinal cancers are the most common and aggressive malignant tumors, accounting for 26% of cancer incidence and 35% of cancer-related mortality worldwide [1]. According to the International Agency for Research on Cancer (IARC), mortality caused by gastrointestinal cancers accounts for 45% of all cancer-related mortality in China [2]. The standard treatment strategies for gastrointestinal cancers include surgery, endoscopy, chemotherapy, radiotherapy, immunotherapy, and targeted therapy [3, 4]. However, disease prognosis remains poor because most patients are diagnosed at an advanced stage. Thus, effective preventive strategies should be implemented to reduce the risk of gastrointestinal cancer.

Studies have found that several diseases could be caused by unhealthy diets, including cancer, and nearly 930,000 cancer-related mortality were induced by poor diet in 2017, especially breast and colorectal cancer (CRC) [5, 6]. The IARC classified red meat as a probable carcinogen based on CRC, pancreatic cancer (PC), and prostate cancer evidence, while processed meat was regarded as carcinogenic to humans based on CRC evidence [7]. Moreover, the World Cancer Research Fund and American Institute for Cancer Research suggest that red meat consumption should be less than three portions per week [8]. Numerous studies have illustrated the relationship between red or processed meat consumption and gastrointestinal cancer [9–12]. However, these studies pooled overall cancer outcomes or focused on a specific type of gastrointestinal cancer, and did not illustrate whether the associations are differing according to study or individuals’ characteristics, including country, sex, follow-up duration, and adjusted level. Thus, the current systematic review and meta-analysis was performed to investigate the associations of red and processed meat consumption with the risk of gastrointestinal cancer, including esophageal cancer (EC), gastric cancer (GC), CRC, colon cancer (CC), rectal cancer (RC), PC, and hepatocellular carcinoma (HCC). Moreover, the exploratory analysis were performed and stratified by country, sex, follow-up duration, and adjusted level.

## Methods

### Data sources, search strategy, and selection criteria

A meta-analysis of observational studies in epidemiology protocols was used for this systematic review and meta-analysis [13]. Prospective cohort studies that assessed the association of red and processed meat consumption with gastrointestinal cancer risk were included in this study, and the publication language and status without restriction. We systematically searched the databases of PubMed, EmBase, and the Cochrane library from their inception until February, 2023, using the following search strategies in PubMed: ((“Red Meat“[Medical Subject Heading (MeSH)]) or (“Meat Products“[MeSH]) or (“processed meat*“[Title/Abstract (tiab)]) or (“hot dog*“[tiab]) or (salami[tiab]) or (pork[tiab]) or (beef[tiab]) or (veal [tiab]) or (sausage[tiab]) or (lamb[tiab]) or (meat*[tiab]) or (bacon[tiab]) or (diet*[tiab])) AND ((“Neoplasms“[Mesh]) or (cancer*[tiab]) or (Neoplasia*[tiab]) or (Neoplasm[tiab]) or (Tumor*[tiab]) or (Tumor *[tiab]) or (Malignan*[tiab]) or (carcinoma[tiab]) or (leukemia[tiab]) or (lymphoma[tiab])). The reference lists of relevant original and review articles were manually reviewed to identify new studies that met the inclusion criteria.

Two reviewers independently performed the literature search and study selection, and conflicts between the reviewers were resolved by a third reviewer who read the full text of the article. Details of the inclusion criteria were as follows: (1) participants: general population; (2) exposure: the highest category of red meat (lamb, mutton, beef, hamburger, and pork), processed meat (sausage and deli meat), or total red and processed meat consumption; (3) control: the lowest category of red meat, processed meat, or total red and processed meat consumption; (4) outcomes: studies that reported at least one of the following outcomes: EC, GC, CRC, CC, RC, PC, and HCC; and (5) study design: studies with a prospective cohort design. For studies that reported several multivariable adjusted effect estimates, we selected the effect estimate that was maximally adjusted for potential confounders. Moreover, if two or more papers reported effect estimates from the same cohort, and the most recently and comprehensive data were obtained.

### Data collection and quality assessment

The two reviewers independently extracted the following information: first authors’ name, study groups’ name, publication year, region, sample size, age, sex, exposure definition, comparisons, follow-up duration, adjusted factors (more than six factors in three parts were considered high; 1–6 factors in 1–2 parts were considered moderate), and reported outcomes. Subsequently, the two reviewers independently assessed the quality of the included studies using the Newcastle-Ottawa Scale (NOS), which contained four items in the selection part, one item in the comparability part, and three items in the outcome part. The “star system” of NOS ranged from 0 to 9, and studies with 7–9 stars were considered as high quality [14]. Inconsistent results regarding data extraction and quality assessment were resolved by a third reviewer who referred to the original article.

### Statistical analysis

The relationship between red meat or processed meat consumption and gastrointestinal cancer risk was assigned as relative risk (RR) with its 95% confidence interval (CI) in individual studies. The random-effects model was used for pooled effect estimates because it considers the underlying variation across the included studies [15, 16]. Heterogeneity among the included studies was assessed using *I*^*2*^ and Q statistics, and significant heterogeneity was defined as *I*^*2*^ ≥ 50.0% or *P* < 0.10 [17, 18]. The robustness of the pooled conclusion was assessed using sensitivity analysis through the sequential removal of a single study [19]. Subgroup analyses were performed to provide exploratory results, which were based on country, sex, follow-up duration, and adjusted level, and the difference between subgroups were compared using the ratio of RRs (RRR) with 95% CIs [20]. Publication bias was assessed using both qualitative and quantitative methods, including funnel plots, Egger’s tests, and Begg’s tests [21, 22]. All reported *P* value were two-sided, and the inspection level was 0.05. The STATA software (version 14.0; Stata Corporation, College Station, TX, USA) was used to perform all statistical analyses.

## Results

### Literature search

**Fig. 1:**
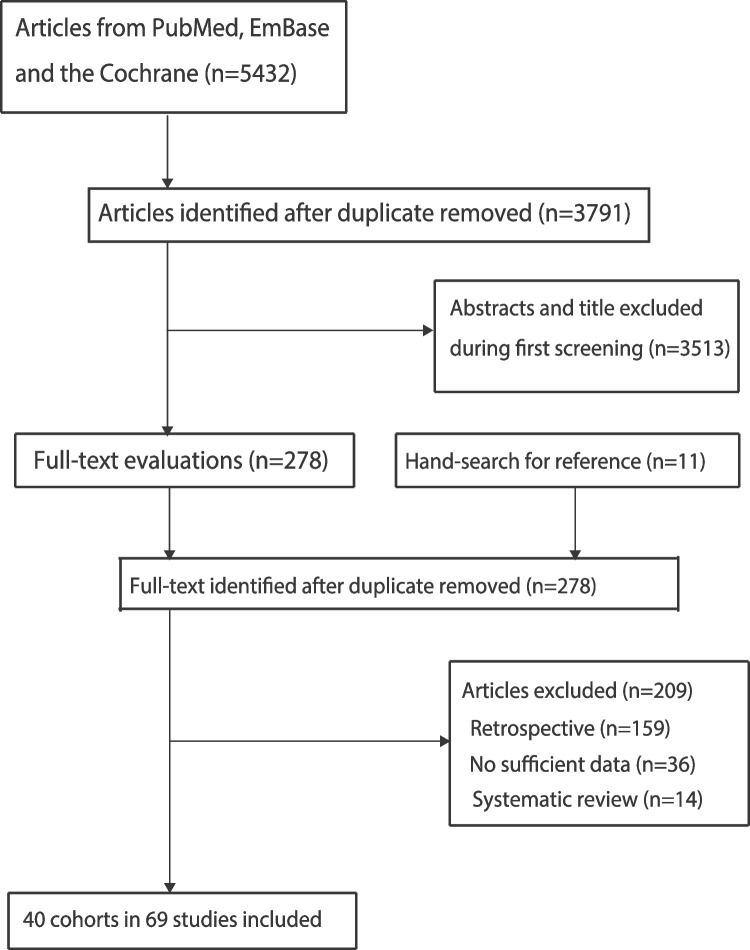
The processes of literature search and study selection

An initial electronic search yielded 5,432 articles. Of these, 3,791 were retained after duplicate titles were removed. After reviewing the titles and abstracts, an additional 3,513 articles were excluded. The remaining 278 articles were retrieved for full-text evaluation, and a total of 40 cohorts reported in 69 articles met the inclusion criteria [23–91]. Review of the reference lists did not yield any new eligible studies. The details of the literature search and the study selection process are shown in Fig. 1.

## Study characteristics

The baseline characteristics of the included studies and the participants are presented in Table 1. A total of 3,780,590 individuals from 40 cohorts were included, and the sample size ranged from 1,583 to 512,891. The follow-up durations of the included studies ranged from 4.1 to 24.0 years. Three cohorts included only male individuals, 11 cohorts included only female individuals, and the remaining 26 cohorts included both male and female individuals. The NOS was applied to assess the quality of the included studies: six cohorts with nine stars, 13 cohorts with eight stars, 14 cohorts with seven stars, and the remaining seven cohorts with six stars.

**Table 1:** The characteristics of included studies and involved participants

Study	Region	Sample size	Age (years)	Sex	Exposure definition	Comparisons	Follow-up (years)	Adjustment factors	Outcome	Study quality
Nomura 1990 (JAH) [23]	USA	7,990	≥ 18	Men and women	FFQ	Quintiles	17.5	Age	GC	6
Bostick 1994 (IWHS) [24, 25]	USA	35,216	55–69	Women	FFQ	Quintiles	5.0	Age, EI, height, parity, vitamin E, vitamin A	CC, PC	8
Kato 1997 (NYUWHS)[26]	USA	14,727	34–65	Women	FFQ	Quartiles	7.1	Age, EI, history of rectal colon polyps	CRC	6
Singh 1998 (AHS) [27]	USA	32,051	≥ 25	Men and women	FFQ	Median	6.0	Age, sex, BMI, PI,parental history of CC, smoking,alcohol, aspirin use	CC	7
Galanis 1998 (JRH) [28]	USA	11,907	≥ 18	Men and women	FFQ	Median	14.8	Age, education, Japanese place of birth, smoking,alcohol	GC	7
Knekt 1999 (FMCHES)[29, 30]	Finland	9,985	15–99	Men and women	Dietary history	Quartiles	24.0	Age, sex, municipality, smoking, EI	CRC, GC, CC, RC	6
Pietinen 1999 (ATBC) [31, 32]	Finland	27,111	50–69	Men	FFQ	Quartiles	8.0	Age, supplement group, smoking, BMI,alcohol,education, PA, calcium intake	CRC, PC	9
Isaksson 2002 (STR) [33]	Sweden	21,884	56	Men and women	FFQ	Tertiles	16.0	Age, sex, smoking	PC	6
Flood 2003 (BCDDP) [34]	USA	45,496	61.9	Women	FFQ	Quintiles	8.5	EI, total meat intake	CRC	8
Michaud 2003 (NHS)[25]	USA	88,802	30–55	Women	FFQ	Quintiles	18.0	Age, smoking, BMI, history of diabetes,EI, height, PA,menopausal status	PC, RC, CC, CRC, HCC	8
Lin 2004 (WHS) [36]	USA	37,547	≥ 45	Women	FFQ	Quintiles	8.7	Age, random treatment assignment, BMI,family history of CRC,history of colorectal polyps, PA, smoking,alcohol, HRT,EI	CRC	8
Wei 2004 (HPFS) [37–40]	USA	46,632	40–75	Men	FFQ	Quintiles	14.0	Age, family history,BMI, PA,alcohol, calcium intake, folate intake,height, smoking,history of endoscopy,beef/pork/lamb as amain dish	RC, CC, CRC, HCC	8
English 2004 (MCCS) [41]	Australia	37,112	27–75	Men and women	FFQ	Quartiles	9.0	Age, sex, country of birth, EI, fat intake, cereal product intake	CRC, RC, CC	9
Chao 2005 (CPS II) [42, 43]	USA	148,610	50–74	Men and women	FFQ	Quintiles	9.0	Age, EI,education, BMI,smoking, PA, multivitamin use, aspirin use, beer,wine,liquor, HRT,fruit intake, vegetable intake, high-fiber grain food intake	RC, CC, PC	9
Larsson 2005 (SMC) [44–46]	Sweden	61,433	40–75	Women	FFQ	Quartiles	13.9	Age, BMI, education, EI, alcohol, saturated fat intake, calcium intake, folate intake, fruit intake, vegetable intake, whole-grain food intake	CRC, RC, GC, PC	7
Norat 2005 (EPIC) [47, 51]	Europe	478,040	21–83	Men and women	FFQ	Quintiles	4.8	Age, sex, center,EI,height, weight,PA,smoking, dietary fiber intake, alcohol intake	CRC, RC, CC, GC, EC, PC, HCC	9
Sauvaget 2005 (LSS) [52]	Japan	38,576	34–98	Men and women	FFQ	Tertiles	20.0	Age, sex, city, radiation dose, smoking, education	GC	7
Nöthlings 2005 (MEC) [53–55]	USA	190,545	45–75	Men and women	FFQ	Quintiles	7.0	Age, sex, ethnicity, history of diabetes,family history of PC,smoking, EI	PC, CRC	8
Berndt 2006 (CLUE II) [56]	USA	1,583	48.5	Men and women	FFQ	Tertiles	13.5	Age, race, EI	CRC	6
Kabat 2007 (CNBSS) [57]	Canada	49,654	40–59	Women	FFQ	Quintiles	16.4	Age, BMI, menopausalstatus, OC use, HRT,dietary fat intake, fiber intake, folic acid intake, EI,smoking, alcohol, education, PA	CRC, RC, CC	7
Cross 2007 (NIH-AARP) [58–63]	USA	494,036	50–71	Men and women	FFQ	Quintiles	8.2	Age, sex, education, marital status, family history of cancer, race, BMI, smoking, PA, EI, alcohol, fruit and vegetable intake	ES, GC, CRC, HCC, PC	9
Butler 2008 (SCHS) [64, 65]	Singapore	61,321	45–74	Men and women	FFQ	Quartiles	10.0	Age, sex, dialect group,interview year, history of diabetes, smoking,BMI, alcohol,education, PA, family history of CRC, EI	CRC, HCC	8
Lee 2009 (SWHS) [66]	China	73,224	40–70	Women	FFQ	Quintiles	7.4	Age, education,income, survey season,tea intake, NSAID use,EI, fiber intake	CRC, RC, CC	8
Heinen 2009 (NLCS) [67-69	Netherlands	3,980	55–69	Men and women	FFQ	Quintiles	13.3	Age, energy intake, sex, smoking, alcohol, history of diabetes, history of hypertension, BMI,vegetable intake, fruit intake	PC, GC, EC, CRC, RC, CC	7
Wie 2014 (CSEC) [70]	Korea	8,024	48.4	Men and women	3-days food records	Median	7.0	Age, sex, EI, BMI, PA,smoking,alcohol use, income,education, marital status	CRC, GC	7
Nomura 2016 (BWHS) [71, 72]	USA	49,103	21–69	Women	FFQ	Tertiles	15.1	Age, geographic regionof residence, EI, smoking, family history of CRC, education,menopausal status,diabetes, insulin use,aspirin use,colonoscopy, sigmoidoscopy, alcohol, BMI	CRC, CC, PC	8
Hastert 2016 (VITAL) [73]	USA	66,920	50–76	Men and women	FFQ	Median	7.6	Age, education,race/ethnicity, receipt of colonoscopy orsigmoidoscopy, family history of CC or RC, NSAID use, history of cancer other than CRC, EI	CRC	7
Jones 2017 (UKWCS) [74]	UK	32,154	52	Women	FFQ	Quintiles	17.4	Age, BMI, EI, PA, smoking,socioeconomic status,family history of CRC	CRC, RC, CC	8
Wada 2017 (Takayama) [75]	Japan	30,331	≥ 35	Men and women	FFQ	Quartiles	16.0	Age, height, BMI,PA,smoking, education,aspirin use, alcohol, fiber intake,calcium intake, vitamin D intake, EI	CRC, RC, CC	7
Pang 2018 (CKB) [76]	China	512,891	30–79	Men and women	FFQ	Median	9.0	Age, sex, study area, education, smoking,alcohol, BMI, PA	PC	8
Diallo 2018 (NSS) [77, 78]	France	61,476	≥ 35	Men and women	24-hour dietary records	Quintiles	4.1	Age, sex, EI,alcohol, number of 24-hour dietary records, smoking,education, PA, height, BMI,family history of cancer, lipids intake,fruit intake, vegetable intake, number of children, red meat intake, processed meat intake	CRC	7
Islam 2019 (pooled 6 studies) [79, 80]	Japan	232,403	40–79	Men and women	FFQ	Quartiles	≥ 10.0	Age, area, history of diabetes, BMI,smoking, alcohol, PA, EI, calcium intake, fiber intake	CRC, RC, CC	7
Mehta 2020 (Sister study) [81]	USA and Puerto Rico	48,704	35–74	Women	FFQ	Quartiles	8.7	EI, BMI,education, PA, race/ethnicity,family history of CRC	CRC, RC, CC	7
Nguyen 2020 (SMHS) [82]	China	60,161	40–74	Men	FFQ	Quartiles	8.1	Sex, age, education,income levels,smoking, alcohol intake,multivitamin use, family history ofCRC, BMI, PA, EI,metabolic condition	CRC, RC, CC	8
Barrubes 2020 (PREDIMED) [83]	Spain	7,216	55–80	Men and women	FFQ	Quartiles	6.0	Age, sex, intervention group, smoking, family history of cancer,education, history of diabetes, EI,aspirin use, weight, PA, plant food intake, fast food and processed food intake, sugar-sweetened beverage intake, alcohol	CRC	6
O’Sullivan 2020 (ATP) [84]	Canada	26,460	50.9	Men and women	Diet history questionnaire	Tertiles	13.2	Age, sex, BMI, fruit and vegetable intake,alcohol, PA, smoking,ethnicity, household income, education,family history of CRC, red or processed meat intake	CRC	7
Mejborn 2020 (DNSDPA) [85]	Denmark	6,282	54.0	Men and women	7-day pre-coded food diaries	Tertiles	10.8	Sex, education,ethnicity, smoking,PA,alcohol, BMI,EI	CRC	6
Zhang 2020 (PLCO) [86]	USA	95,962	55–74	Men and women	Diet history questionnaire	Tertiles	8.9	Age, sex, race, education, smoking, aspirin use, history of diabetes, family history of PC, EI,PA, weight, diet rich in whole grains,vegetables, fruit, and beans, ultra-processed food intake, sugar-sweetened drink intake,alcohol, breastfeeding	PC	9
Knuppel 2020 (UK Biobank) [87–90]	UK	474,996	37–73	Men and women	FFQ	Quintiles	6.9	Age, region, ethnicity, deprivation,qualification, employment, living with spouse/partner, height, smoking, PA, alcohol, fruit and vegetable intake, cereal fiber intake, cheese intake,milk added to tea/coffee/cereal, oily fish intake, non-oily fish intake, menopausal status, parity, HRT, OC use	EC, GC, CRC, CC, RC, HCC, PC	8
Collatuzzo 2022 (GCS) [91]	Iran	50,045	40–75	Men and women	FFQ	Quintiles	12.0	Age, sex, BMI, ethnicity, place of residence, education and hot tea consumption	PC, EC, GC	7

*AHS Adventist Health Study, *ATBC *Alpha-Tocopherol, Beta-Carotene Cancer Prevention Study, *ATP *Alberta’s Tomorrow Project, *BCDDP *Breast Cancer Detection Demonstration Project, *BMI *Body mass index, *CC *Colon cancer, *CKB *China Kadoorie Biobank, *CNBSS *Canadian National BreastScreening Study, *CRC *Colorectal cancer, *CSEC *CancerScreening Examination Cohort of the National Cancer Centerof Korea, *EI *Energy intake, *EPIC *European Prospective Investigation into Cancerand Nutrition, *FFQ *Food-frequency questionnaire, *GC *Gastric cancer, *GCS *Golestan Cohort Study, *HPFS *Health Professionals Follow Up Study, *HRT *Hormone replacement therapy, *IWHS *Iowa Women’s Health Study, *JAH *Japanese Ancestry in Hawaii, *JRH *Japanese residents of Hawaii, *LSS *Life Span Study, *MCCS *Melbourne Collaborative Cohort Study, *MEC *Multiethnic Cohort Study, *NHS *Nurses’ Health Study, *NIH-AARP *National Institutes of Health-American Association for Retired Persons, *NLCS *Netherlands Cohort Study, *NSS *NutriNet-Sante Study, *NYUWHS *New York University Women’s Health Study, *PA *Physical activity, *PC *Pancreatic cancer, *PLCO *Prostate, Lung, Colorectal, and Ovarian Cancer Screening Trial, *PREDIMED *Spanish PREvencion con DIeta MEDiterranea, *RC *Rectal cancer, *SCHS * Singapore Chinese Health Study, *SMC *Swedish Mammography Cohort, *STR *Swedish Twin Registry, *SWHS *Shanghai Women’s Health Study, *UKWCS *UK Women’s Cohort Study, *VITAL *VITamins And Lifestyle, *WHS *Women’s Health Study

### EC

**Fig. 2:**
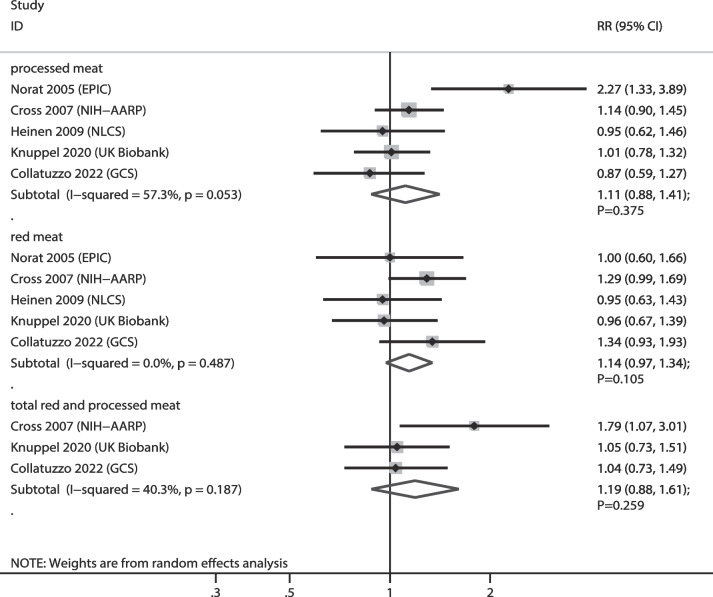
Association of meat consumption with the risk of esophageal cancer. RR: relative risk; CI: confidence interval

The numbers of cohorts that reported the associations of red meat, processed meat, and total red and processed meat consumption with EC risk were 5, 5, and 3 cohorts, respectively. The summary results indicated that higher consumption of red meat (RR: 1.14; 95% CI: 0.97–1.34; *P* = 0.105), processed meat (RR: 1.11; 95% CI: 0.88–1.41; *P* = 0.375), and total red and processed meat (RR: 1.19; 95% CI: 0.88–1.61; *P* = 0.259) were not associated with the risk of EC (Fig. 2). Moreover, we noted a significant heterogeneity in the relationship between processed meat consumption and EC (*I*^*2*^ = 57.3%; *P* = 0.053). Sensitivity analyses revealed that the pooled conclusions for the relationship between red meat, processed meat, and total red and processed meat consumption and EC risk were robust (Supplementary file 1). The results of the subgroup analyses were consistent with those of the overall analyses, and the results showed no significant associations (Table 2). There was no significant publication bias for red (*P* value for Egger: 0.230; *P* value for Begg: 0.806) and processed meat (*P* value for Egger: 0.540; *P* value for Begg: 0.806) consumption, whereas there was a significant publication bias for total red and processed meat consumption (*P* value for Egger: 0.018; *P* value for Begg: 0.296) (Supplementary file 2).

**Table 2:** Subgroup analyses for the association between meat consumption and the risk of gastrointestinal cancer

Outcomes	Exposure	Factors	Subgroups	RR and 95%CI	*P* value	Heterogeneity (%)	RRR between subgroups
EC	Red meat	Country	Western	1.10 (0.87–1.40)	0.428	33.4	0.82 (0.53–1.27)^a^
			Eastern	1.34 (0.93–1.93)	0.116	-	
		Sex	Male	1.35 (0.57–3.20)	0.496	75.7	1.23 (0.50–3.03)^b^
			Female	1.10 (0.84–1.45)	0.496	0.0	
		Follow-up	≥ 10.0	1.10 (0.72–1.68)	0.662	45.8	0.96 (0.59–1.55)^c^
			< 10.0	1.15 (0.91–1.45)	0.252	25.6	
		Adjusted level	High	1.10 (0.87–1.40)	0.428	33.4	0.82 (0.53–1.27)^d^
			Moderate	1.34 (0.93–1.93)	0.116	-	
	Processed meat	Country	Western	1.17 (0.89–1.55)	0.262	56.7	1.34 (0.84–2.17)^a^
			Eastern	0.87 (0.59–1.28)	0.476	-	
		Sex	Male	1.44 (0.81–2.58)	0.218	51.3	1.62 (0.80–3.26)^b^
			Female	0.89 (0.60–1.32)	0.563	26.3	
		Follow-up	≥ 10.0	0.94 (0.60–1.49)	0.798	49.3	0.75 (0.44–1.29)^c^
			< 10.0	1.25 (0.94–1.67)	0.127	60.3	
		Adjusted level	High	1.17 (0.89–1.55)	0.262	56.7	1.34 (0.84–2.17)^d^
			Moderate	0.87 (0.59–1.28)	0.476	-	
	Total red and processed meat	Country	Western	1.33 (0.79–2.23)	0.286	63.5	1.28 (0.68–2.40)^a^
			Eastern	1.04 (0.73–1.49)	0.829	-	
		Sex	Male	-	-	-	-
			Female	-	-	-	
		Follow-up	≥ 10.0	1.04 (0.73–1.49)	0.829	-	0.78 (0.42–1.47)^c^
			< 10.0	1.33 (0.79–2.23)	0.286	63.5	
		Adjusted level	High	1.33 (0.79–2.23)	0.286	63.5	1.28 (0.68–2.40)^d^
			Moderate	1.04 (0.73–1.49)	0.829	-	
GC	Red meat	Country	Western	1.02 (0.86–1.21)	0.805	24.7	1.00 (0.80–1.25)^a^
			Eastern	1.02 (0.88–1.17)	0.818	31.1	
		Sex	Male	1.01 (0.87–1.17)	0.885	0.0	1.12 (0.88–1.43)^b^
			Female	0.90 (0.74–1.09)	0.268	0.0	
		Follow-up	≥ 10.0	1.01 (0.91–1.12)	0.869	0.0	0.97 (0.72–1.32)^c^
			< 10.0	1.04 (0.78–1.38)	0.792	55.7	
		Adjusted level	High	0.98 (0.88–1.10)	0.758	12.2	0.84 (0.64–1.12)^d^
			Moderate	1.16 (0.90–1.50)	0.250	31.6	
	Processed meat	Country	Western	1.11 (0.94–1.33)	0.221	42.4	1.12 (0.90–1.40)^a^
			Eastern	0.99 (0.86–1.13)	0.871	14.0	
		Sex	Male	1.02 (0.88–1.18)	0.774	0.0	0.89 (0.62–1.27)^b^
			Female	1.15 (0.83–1.59)	0.404	62.1	
		Follow-up	≥ 10.0	1.08 (0.94–1.25)	0.276	35.3	1.03 (0.74–1.42)^c^
			< 10.0	1.05 (0.78–1.40)	0.759	61.0	
		Adjusted level	High	1.07 (0.92–1.24)	0.382	50.4	0.97 (0.72–1.31)^d^
			Moderate	1.10 (0.85–1.42)	0.479	20.9	
	Total red and processed meat	Country	Western	0.97 (0.76–1.24)	0.804	8.6	0.98 (0.72–1.34)^a^
			Eastern	0.99 (0.81–1.20)	0.896	45.6	
		Sex	Male	0.96 (0.82–1.13)	0.618	-	1.17 (0.88–1.55)^b^
			Female	0.82 (0.65–1.03)	0.091	-	
		Follow-up	≥ 10.0	0.98 (0.78–1.23)	0.873	61.9	0.99 (0.72–1.35)^c^
			< 10.0	0.99 (0.80–1.23)	0.927	0.0	
		Adjusted level	High	0.93 (0.83–1.04)	0.221	0.0	**0.68 (0.46-1.00)** ^d^
			Moderate	1.37 (0.94–1.99)	0.100	-	
CRC	Red meat	Country	Western	**1.12 (1.04–1.19)**	**0.001**	20.4	1.11 (0.98–1.26)^a^
			Eastern	1.01 (0.91–1.13)	0.830	0.0	
		Sex	Male	0.97 (0.80–1.18)	0.789	0.0	0.92 (0.75–1.14)^b^
			Female	1.05 (0.96–1.15)	0.298	0.0	
		Follow-up	≥ 10.0	1.06 (0.98–1.15)	0.131	0.0	0.96 (0.84–1.11)^c^
			< 10.0	1.10 (0.98–1.23)	0.092	54.4	
		Adjusted level	High	**1.08 (1.01–1.15)**	**0.020**	28.0	0.86 (0.68–1.10)^d^
			Moderate	1.25 (0.99–1.59)	0.058	0.0	
	Processed meat	Country	Western	**1.20 (1.13–1.29)**	**< 0.001**	19.6	1.02 (0.89–1.16)^a^
			Eastern	**1.18 (1.06–1.32)**	**0.003**	0.0	
		Sex	Male	**1.27 (1.08–1.50)**	**0.005**	23.8	1.11 (0.91–1.36)^b^
			Female	**1.14 (1.02–1.28)**	**0.023**	0.0	
		Follow-up	≥ 10.0	**1.21 (1.12–1.32)**	**< 0.001**	0.0	1.03 (0.91–1.05)^c^
			< 10.0	**1.18 (1.09–1.29)**	**< 0.001**	25.7	
		Adjusted level	High	**1.18 (1.12–1.24)**	**< 0.001**	0.0	0.91 (0.71–1.16)^d^
			Moderate	**1.30 (1.03–1.66)**	**0.030**	43.0	
	Total red and processed meat	Country	Western	**1.15 (1.07–1.23)**	**< 0.001**	1.5	1.11 (0.95–1.28)^a^
			Eastern	1.04 (0.91–1.18)	0.574	0.0	
		Sex	Male	1.21 (0.98–1.49)	0.070	0.0	1.09 (0.85–1.40)^b^
			Female	1.11 (0.97–1.26)	0.128	15.4	
		Follow-up	≥ 10.0	**1.20 (1.05–1.37)**	**0.007**	0.0	1.08 (0.92–1.27)^c^
			< 10.0	**1.11 (1.02–1.22)**	**0.016**	23.3	
		Adjusted level	High	**1.13 (1.05–1.21)**	**0.001**	10.9	0.97 (0.76–1.23)^d^
			Moderate	1.17 (0.92–1.47)	0.195	0.0	
CC	Red meat	Country	Western	**1.18 (1.03–1.35)**	**0.015**	7.2	1.10 (0.91–1.34)^a^
			Eastern	1.07 (0.93–1.24)	0.346	0.0	
		Sex	Male	1.05 (0.78–1.41)	0.747	-	1.02 (0.73–1.42)^b^
			Female	1.03 (0.89–1.19)	0.675	0.0	
		Follow-up	≥ 10.0	1.05 (0.92–1.20)	0.443	0.0	0.85 (0.71–1.03)^c^
			< 10.0	**1.23 (1.08–1.42)**	**0.003**	0.0	
		Adjusted level	High	**1.14 (1.02–1.27)**	**0.021**	13.0	1.07 (0.75–1.52)^d^
			Moderate	1.07 (0.76–1.50)	0.708	0.0	
	Processed meat	Country	Western	**1.24 (1.12–1.38)**	**< 0.001**	0.0	0.99 (0.80–1.23)^a^
			Eastern	**1.25 (1.03–1.50)**	**0.022**	0.0	
		Sex	Male	**1.24 (1.04–1.49)**	**0.018**	0.0	0.99 (0.78–1.26)^b^
			Female	**1.25 (1.06–1.47)**	**0.007**	0.0	
		Follow-up	≥ 10.0	**1.25 (1.09–1.44)**	**0.002**	0.0	1.01 (0.84–1.22)^c^
			< 10.0	**1.24 (1.09–1.40)**	**0.001**	0.0	
		Adjusted level	High	**1.22 (1.11–1.35)**	**< 0.001**	0.0	0.87 (0.64–1.17)^d^
			Moderate	**1.41 (1.06–1.88)**	**0.019**	0.0	
	Total red and processed meat	Country	Western	**1.24 (1.10–1.41)**	**0.001**	0.0	1.06 (0.79–1.42)^a^
			Eastern	1.17 (0.90–1.52)	0.239	62.3	
		Sex	Male	**1.28 (1.02–1.61)**	**0.032**	0.0	1.12 (0.84–1.51)^b^
			Female	1.14 (0.95–1.38)	0.158	4.1	
		Follow-up	≥ 10.0	**1.26 (1.05–1.51)**	**0.014**	0.0	1.10 (0.85–1.40)^c^
			< 10.0	1.15 (0.97–1.36)	0.115	50.1	
		Adjusted level	High	**1.19 (1.04–1.35)**	**0.012**	40.0	1.07 (0.67–1.71)^d^
			Moderate	1.11 (0.71–1.74)	0.635	0.0	
RC	Red meat	Country	Western	**1.33 (1.03–1.72)**	**0.029**	44.5	1.41 (0.95–2.11)^a^
			Eastern	0.94 (0.69–1.27)	0.678	22.4	
		Sex	Male	1.21 (0.67–2.18)	0.527	-	1.07 (0.54–2.13)^b^
			Female	1.13 (0.79–1.61)	0.518	59.8	
		Follow-up	≥ 10.0	1.23 (0.94–1.60)	0.134	35.6	1.07 (0.68–1.69)^c^
			< 10.0	1.15 (0.79–1.66)	0.467	58.8	
		Adjusted level	High	1.10 (0.91–1.33)	0.332	32.4	**0.50 (0.28–0.91)** ^d^
			Moderate	**2.18 (1.25–3.81)**	**0.006**	0.0	
	Processed meat	Country	Western	**1.29 (1.07–1.56)**	**0.007**	19.9	1.13 (0.84–1.53)^a^
			Eastern	1.14 (0.90–1.44)	0.277	0.0	
		Sex	Male	1.03 (0.79–1.36)	0.824	0.0	0.94 (0.65–1.38)^b^
			Female	1.09 (0.84–1.43)	0.515	6.5	
		Follow-up	≥ 10.0	1.09 (0.91–1.31)	0.345	0.0	**0.77 (0.60-1.00)** ^c^
			< 10.0	**1.41 (1.17–1.69)**	**< 0.001**	0.0	
		Adjusted level	High	**1.20 (1.05–1.37)**	**0.007**	0.0	0.63 (0.38–1.06)^d^
			Moderate	**1.90 (1.15–3.13)**	**0.012**	0.0	
	Total red and processed meat	Country	Western	**1.32 (1.11–1.57)**	**0.002**	0.0	1.26 (0.98–1.61)^a^
			Eastern	1.05 (0.88–1.26)	0.587	0.0	
		Sex	Male	0.93 (0.58–1.49)	0.763	-	0.84 (0.48–1.46)^b^
			Female	1.11 (0.83–1.49)	0.478	0.0	
		Follow-up	≥ 10.0	1.09 (0.85–1.39)	0.507	0.0	0.85 (0.61–1.18)^c^
			< 10.0	**1.28 (1.03–1.60)**	**0.025**	44.9	
		Adjusted level	High	**1.19 (1.03–1.37)**	**0.021**	14.3	0.65 (0.21-2.00)^d^
			Moderate	1.82 (0.60–5.52)	0.290	-	
PC	Red meat	Country	Western	1.03 (0.87–1.21)	0.764	55.0	1.91 (0.87–4.18)^a^
			Eastern	0.54 (0.25–1.16)	0.116	-	
		Sex	Male	0.89 (0.70–1.14)	0.360	0.0	0.82 (0.58–1.15)^b^
			Female	1.09 (0.86–1.38)	0.494	33.3	
		Follow-up	≥ 10.0	0.94 (0.61–1.44)	0.777	63.6	0.91 (0.57–1.45)^c^
			< 10.0	1.03 (0.86–1.23)	0.779	55.2	
		Adjusted level	High	1.03 (0.86–1.22)	0.770	59.5	1.37 (0.73–2.58)^d^
			Moderate	0.75 (0.41–1.38)	0.357	27.4	
	Processed meat	Country	Western	0.99 (0.83–1.18)	0.925	68.7	0.85 (0.43–1.68)^a^
			Eastern	1.16 (0.60–2.23)	0.657	-	
		Sex	Male	0.99 (0.76–1.30)	0.953	59.2	1.09 (0.78–1.51)^b^
			Female	0.91 (0.75–1.09)	0.293	28.5	
		Follow-up	≥ 10.0	0.88 (0.71–1.10)	0.275	0.0	0.84 (0.62–1.13)^c^
			< 10.0	1.05 (0.86–1.29)	0.639	74.1	
		Adjusted level	High	0.99 (0.83–1.17)	0.870	68.2	0.98 (0.46–2.09)^d^
			Moderate	1.01 (0.48–2.10)	0.984	69.5	
	Total red and processed meat	Country	Western	1.04 (0.87–1.24)	0.699	43.4	0.80 (0.62–1.03)^a^
			Eastern	**1.30 (1.09–1.56)**	**0.004**	-	
		Sex	Male	0.86 (0.65–1.14)	0.287	0.0	0.96 (0.65–1.40)^b^
			Female	0.90 (0.70–1.17)	0.439	0.0	
		Follow-up	≥ 10.0	1.06 (0.72–1.56)	0.761	0.0	0.97 (0.64–1.49)^c^
			< 10.0	1.09 (0.91–1.30)	0.363	59.3	
		Adjusted level	High	1.08 (0.93–1.27)	0.305	48.3	-
			Moderate	-	-	-	
HCC	Red meat	Country	Western	1.04 (0.99–1.09)	0.103	0.0	0.90 (0.68–1.19)^a^
			Eastern	1.16 (0.88–1.53)	0.299	-	
		Sex	Male	1.02 (0.97–1.08)	0.442	0.0	0.92 (0.81–1.04)^b^
			Female	1.11 (1.00-1.24)	0.055	0.0	
		Follow-up	≥ 10.0	1.13 (0.89–1.43)	0.315	0.0	1.09 (0.85–1.38)^c^
			< 10.0	1.04 (0.99–1.09)	0.106	0.0	
		Adjusted level	High	1.04 (1.00-1.09)	0.075	0.0	-
			Moderate	-	-	-	
	Processed meat	Country	Western	0.97 (0.85–1.10)	0.647	50.0	0.84 (0.63–1.13)^a^
			Eastern	1.15 (0.89–1.49)	0.288	-	
		Sex	Male	1.37 (0.54–3.46)	0.502	86.1	1.32 (0.49–3.53)^b^
			Female	1.04 (0.74–1.44)	0.837	37.7	
		Follow-up	≥ 10.0	1.43 (0.96–2.13)	0.081	47.2	**1.55 (1.04–2.32)** ^c^
			< 10.0	**0.92 (0.87–0.97)**	**0.002**	0.0	
		Adjusted level	High	1.00 (0.88–1.13)	0.979	52.3	-
			Moderate	-	-	-	
	Total red and processed meat	Country	Western	0.94 (0.67–1.32)	0.724	20.9	0.74 (0.48–1.15)^a^
			Eastern	1.27 (0.96–1.68)	0.094	-	
		Sex	Male	1.69 (0.74–3.87)	0.214	-	1.71 (0.58–5.06)^b^
			Female	0.99 (0.49-2.00)	0.978	-	
		Follow-up	≥ 10.0	1.26 (0.99–1.62)	0.065	0.0	1.54 (0.96–2.45)^c^
			< 10.0	0.82 (0.55–1.22)	0.324	23.2	
		Adjusted level	High	1.05 (0.80–1.39)	0.709	34.1	-
			Moderate	-	-	-	

*^a^compared Western countries with Eastern countries; ^b^compared male with female; ^c^compared follow-up ≥ 10.0 years with follow-up < 10.0 years; ^d^compared high adjusted level with moderate adjusted level

### GC

**Fig. 3:**
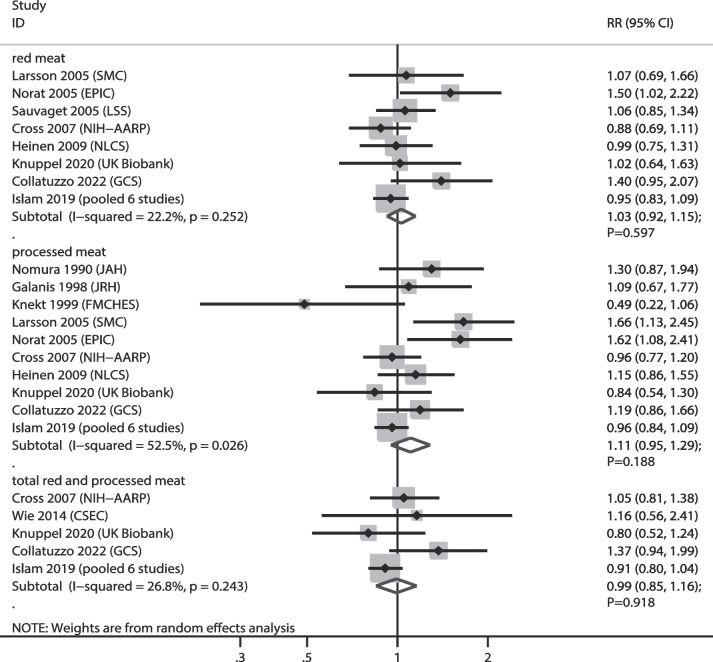
Association of meat consumption with the risk of gastric cancer. RR: relative risk; CI: confidence interval

The numbers of cohorts that reported the associations of red meat, processed meat, and total red and processed meat consumptions with GC risk were 8, 10, and 5 cohorts, respectively. We noted that higher consumption of red meat (RR: 1.03; 95% CI: 0.92–1.15; *P* = 0.597), processed meat (RR: 1.11; 95% CI: 0.95–1.29; *P* = 0.188), and total red and processed meat (RR: 0.99; 95% CI: 0.85–1.16; *P* = 0.918) were not associated with GC risk (Fig. 3). There was a significant heterogeneity in the relationship between processed meat consumption and GC (*I*^*2*^ = 52.5%; *P* = 0.026). Sensitivity analyses indicated that the associations between red meat, processed meat, and total red and processed meat consumption with GC risk were stable, and no significant associations were observed (Supplementary file 1). Subgroup analyses indicated no significant association between red meat, processed meat, and total red and processed meat with GC risk in all subsets (Table 2). No significant publication bias was observed for red meat (*P* value for Egger: 0.095; *P* value for Begg: 0.536), processed meat (*P* value for Egger: 0.395; *P* value for Begg: 1.000), and total red and processed meat consumption (*P* value for Egger: 0.388; *P* value for Begg: 0.806) (Supplementary file 2).

### CRC

**Fig. 4:**
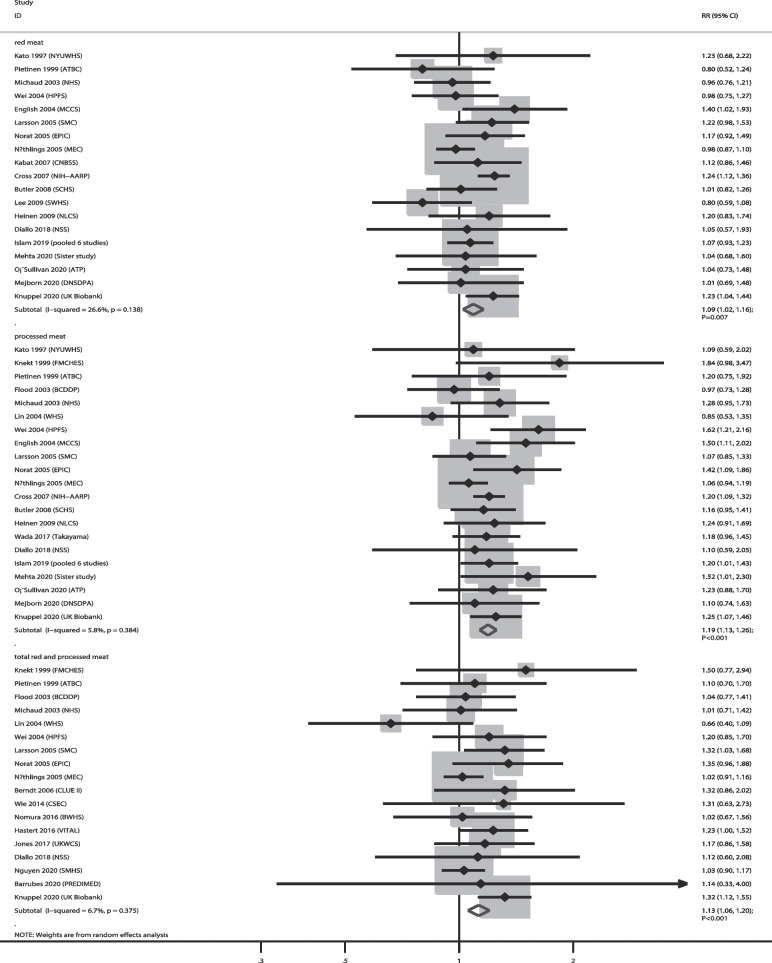
Association of meat consumption with the risk of colorectal cancer. RR: relative risk; CI: confidence interval

The numbers of cohorts that reported the associations of red meat, processed meat, and total red and processed meat consumption with CRC risk were 19, 21, and 18, respectively. The summary results indicated that higher consumption of red meat (RR: 1.09; 95% CI: 1.02–1.16; *P* = 0.007), processed meat (RR: 1.19; 95% CI: 1.13–1.26; *P* < 0.001), and total red and processed meat (RR: 1.13; 95% CI: 1.06–1.20; *P* < 0.001) were associated with an increased risk of CRC, and no significant heterogeneity was observed across the included studies (Fig. 4). Sensitivity analysis indicated that the pooled conclusions regarding the relationship between red meat, processed meat, and total red and processed meat consumption with GC risk were not altered by the sequential removal of a single study (Supplementary file 1). Subgroup analyses revealed that higher red meat consumption was associated with an increased risk of CRC when pooled studies were conducted in Western countries and studies with high adjusted levels; higher processed meat consumption was associated with an increased risk of CRC in all subgroups, and higher total red and processed meat consumption was associated with an increased risk of CRC when pooled studies were conducted in Western countries, irrespective of follow-up duration and studies with high adjusted levels (Table 2). There was no significant publication bias for red meat (*P* value for Egger: 0.302; *P* value for Begg: 0.726), processed meat (*P* value for Egger: 0.305; *P* value for Begg: 0.928), and total red and processed meat consumption (*P* value for Egger: 0.511; *P* value for Begg: 1.000) (Supplementary file 2).

### CC

**Fig. 5:**
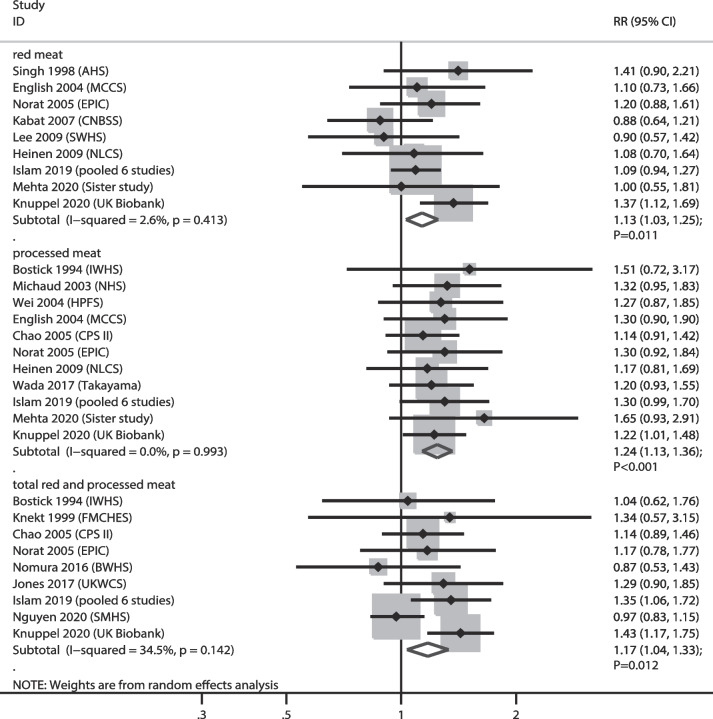
Association of meat consumption with the risk of colon cancer. RR: relative risk; CI: confidence interval

The numbers of cohorts that reported the associations of red meat, processed meat, and total red and processed meat consumption with the risk of CC were 9, 11, and 9 cohorts, respectively. We noted that higher consumption of red meat (RR: 1.13; 95% CI: 1.03–1.25; *P* = 0.011), processed meat (RR: 1.24; 95% CI: 1.13–1.36; *P* < 0.001), and total red and processed meat (RR: 1.17; 95% CI: 1.04–1.33; *P* = 0.012) were associated with an increased risk of CC, and no significant heterogeneity was observed across the included studies (Fig. 5). Sensitivity analyses indicated that the pooled conclusions for the relationship between red meat and total red and processed meat consumption with CC risk were variables with marginal 95% CI (Supplementary file 1). Subgroup analyses indicated that higher red meat consumption was associated with an increased risk of CC when pooled studies were conducted in Western countries, follow-up < 10.0 years, and studies with high adjusted levels. The relationship of processed meat consumption with the risk of CC was statistically significant in all subgroups; high total red and processed meat consumption was associated with an increased risk of CC when pooled studies were conducted in Western countries, male sex, follow-up ≥ 10.0 years, and studies with high adjusted levels (Table 2). We noted no significant publication bias for red meat (*P* value for Egger: 0.602; *P* value for Begg: 0.602), and total red and processed meat consumption (*P* value for Egger: 0.879; *P* value for Begg: 0.602), whereas a significant publication bias was observed for processed meat consumption (*P* value for Egger: 0.010; *P* value for Begg: 0.119) (Supplementary file 2).

### RC

**Fig. 6:**
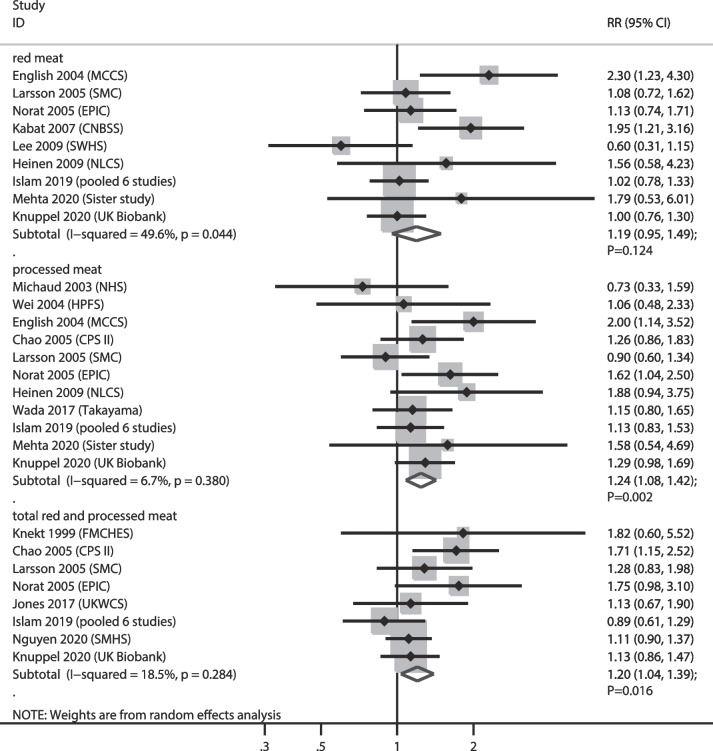
Association of meat consumption with the risk of rectal cancer. RR: relative risk; CI: confidence interval

The numbers of cohorts that reported the associations of red meat, processed meat, and total red and processed meat consumption with the risk of RC were 9, 11, and 8 cohorts, respectively. We noted that higher consumption of processed meat (RR: 1.24; 95% CI:–1.08–1.42; *P* = 0.002) and total red and processed meat (RR: 1.20; 95% CI: 1.04–1.39; *P* = 0.016) were associated with an increased risk of RC, while red meat consumption was not associated with the risk of RC (RR: 1.19; 95% CI: 0.95–1.49; *P* = 0.124). Moreover, we noted significant heterogeneity in the relationship between red meat consumption and RC among the included studies (*I*^*2*^ = 49.6%; *P* = 0.044) (Fig. 6). Sensitivity analyses indicated that the pooled conclusions for the relationship between red meat and total red and processed meat consumption and the risk of RC were variable (Supplementary File 1). Subgroup analyses revealed that higher consumption of red meat intake was associated with an increased risk of RC when pooled studies were conducted in Western countries, and pooled studies with moderately adjusted levels, and the strength of relation in the subgroups of studies with higher adjusted levels was significantly lower than those of studies with moderate adjusted levels (RRR: 0.50; 95% CI: 0.28–0.91). Furthermore, higher processed meat consumption was associated with an increased risk of RC when pooled studies were conducted in Western countries, follow-up duration < 10.0 years, and irrespective of the adjusted level, while the strength of the relationship in the subgroup with longer follow-up duration was significantly lower than that in the subgroup with shorter follow-up duration (RRR: 0.77; 95% CI: 0.60–1.00) (Table 2). There was no significant publication bias for red meat (*P* value for Egger: 0.258; *P* value for Begg: 0.251), processed meat (*P* value for Egger: 0.657; *P* value for Begg: 0.640), and total red and processed meat consumption (*P* value for Egger: 0.208; *P* value for Begg: 0.174) (Supplementary file 2).

### PC

**Fig. 7:**
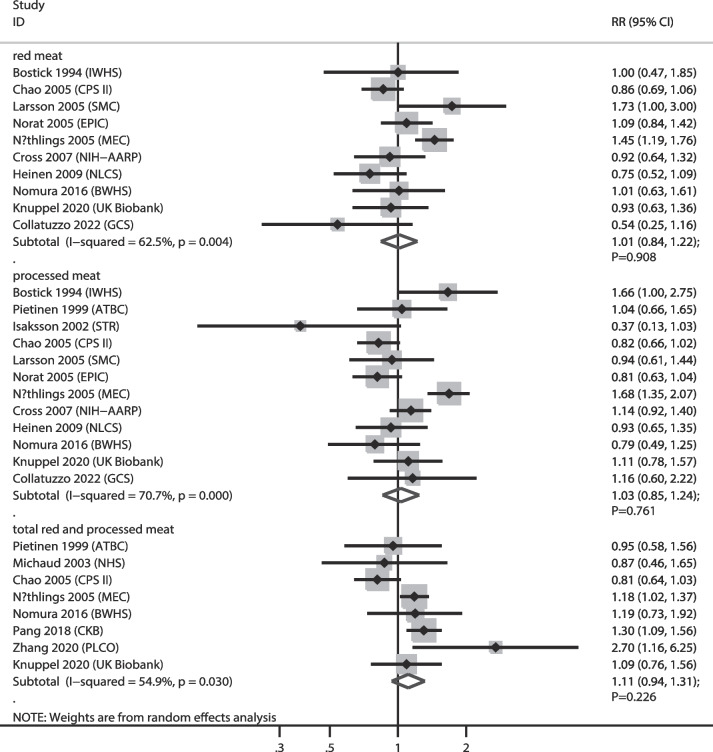
Association of meat consumption with the risk of pancreatic cancer. RR: relative risk; CI: confidence interval

The numbers of cohorts that reported the associations of red meat, processed meat, and total red and processed meat consumption with PC risk were 10, 12, and 8 cohorts, respectively. The summary results indicated that higher consumption of red meat (RR: 1.01; 95% CI: 0.84–1.22; *P* = 0.908), processed meat (RR: 1.03; 95% CI: 0.85–1.24; *P* = 0.761), and total red and processed meat (RR: 1.11; 95% CI: 0.94–1.31; *P* = 0.226) were not associated with PC risk, and significant heterogeneity was observed for red meat (*I*^*2*^ = 62.5%; *P* = 0.004), processed meat (*I*^*2*^ = 70.7%; *P* < 0.001), and total red and processed meat consumption (*I*^*2*^ = 54.9%; *P* = 0.030) (Fig. 7). Sensitivity analysis indicated that higher total red and processed meat consumption were associated with an increased risk of PC after removing the CPS II cohort [42, 43] (Supplementary file 1). Subgroup analyses revealed that total red and processed meat consumption were associated with an increased risk of PC when pooled studies were conducted in Eastern countries (Table 2). No significant publication bias for red meat (*P* value for Egger: 0.365; *P* value for Begg: 1.000), processed meat (*P* value for Egger: 0.458; *P* value for Begg: 0.945), and total red and processed meat consumption (*P* value for Egger: 0.928; *P* value for Begg: 0.902) was observed (Supplementary file 2).

### HCC

**Fig. 8:**
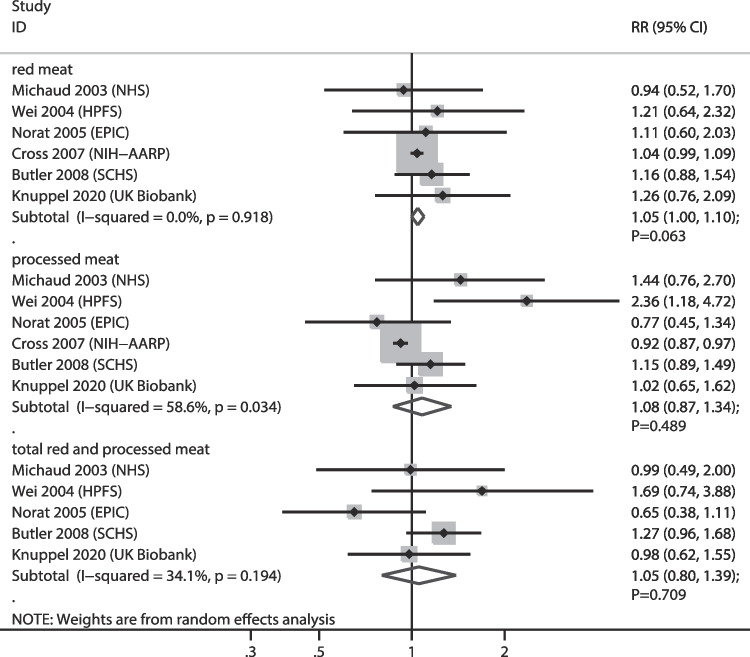
Association of meat consumption with the risk of hepatocellular carcinoma. RR: relative risk; CI: confidence interval

The numbers of cohorts that reported the associations of red meat, processed meat, and total red and processed meat consumption with HCC risk were 6, 6, and 5 cohorts, respectively. Red meat (RR: 1.05; 95% CI: 1.00–1.10; *P* = 0.063), processed meat (RR: 1.08; 95% CI: 0.87–1.34; *P* = 0.489), and total red and processed meat consumption (RR: 1.05; 95% CI: 0.80–1.39; *P* = 0.709) were not associated with HCC risk, and significant heterogeneity was observed for the relationship between processed meat consumption and HCC (*I*^*2*^ = 58.6%; *P* = 0.034) (Fig. 8). Sensitivity analysis indicated that higher red meat consumption was associated with an increased risk of HCC after removing the NHS cohort [35] (Supplementary file 1). Subgroup analyses revealed that processed meat consumption was associated with a reduced risk of HCC when the follow-up duration was < 10.0 years, and the strength for the subgroup of follow-up ≥ 10.0 years was greater than that of the follow-up < 10.0 years subgroup (RRR: 1.55; 95% CI: 1.04–2.32). There was no significant publication bias for red meat (*P* value for Egger: 0.170; *P* value for Begg: 1.000), processed meat (*P* value for Egger: 0.133; *P* value for Begg: 0.260), and total red and processed meat consumption (*P* value for Egger: 0.649; *P* value for Begg: 1.000) (Supplementary file 2).

## Discussion

This systematic review and meta-analysis identified 40 cohorts and involved a total of 3,780,590 individuals with a wide range of characteristics. This study found that higher consumption of processed meat and total red and processed meat was associated with the risk of developing CRC, CC, and RC, while red meat was associated with an increased risk of developing CRC and CC. However, meat intake was not associated with the risk of EC, GC, PC, or HCC. Sensitivity analysis indicated that higher total red and processed meat consumption might increase risk of PC, and higher red meat consumption might cause an additional risk of HCC. Subgroup analyses revealed that the strength of higher consumption of total red and processed meat with the risk of GC in subgroup of high adjusted level was lower than subgroup of moderate adjusted level. Moreover, the strength of higher consumption of red meat with the risk of RC in subgroup of high adjusted level was lower than subgroup of moderate adjusted level, while the strength of higher consumption of processed meat with the risk of RC in subgroup of follow-up ≥ 10.0 years was lower than subgroup of follow-up < 10.0 years. Finally, the strength of higher consumption of processed meat with the risk of HCC in subgroup of follow-up ≥ 10.0 years was higher than subgroup of follow-up < 10.0 years.

Several systematic reviews and meta-analyses have illustrated the association between meat consumption and the risk of gastrointestinal cancer [9–12]. Yu et al. identified 17 observational studies and found that higher processed meat consumption was associated with an increased risk of HCC, while the risk of HCC was reduced in individuals who consumed higher amounts of white meat and fish [9]. Farvid et al. identified 148 studies and found that higher red meat consumption was associated with an increased risk of CRC, CC, RC, and HCC, while processed meat consumption was associated with an increased risk of CRC, CC, and RC. Moreover, they pointed out that total red and processed meat consumption were associated with an increased risk of CRC, CC, and RC [10]. Händel et al. identified 29 prospective cohort studies and found that high versus low processed meat consumption was associated with an increased risk of CRC, CC, and RC, and dose-response analysis reported similar outcomes [11]. Han et al. identified 56 cohorts and suggested that the absolute effects of red and processed meat consumption on cancer incidence are small, whereas reduction of processed meat is associated with a reduced risk of EC and CRC [12]. The current updated systematic review and meta-analysis was performed to assess the strength of the relationship between meat consumption and gastrointestinal cancer risk. Moreover, an exploratory analysis were performed according to the study or individual characteristics, including country, sex, follow-up duration, and adjusted level.

The summary of our results indicated higher red meat, processed meat, and total red and processed meat did not affect the risk of EC, which was inconsistent with prior meta-analysis [92]. Previous studies reported that red and processed meat consumption were associated with an increased risk of EC [92], and explained this by the high amounts of heme iron and N-glycolylneuraminic acid contained in red meat, which could catalyze lipid peroxidation and DNA damage, and potentially immunogenic molecules could induce tumors [93–95]. Moreover, processed meat contains high amounts of saturated fats, which play an important role in the risk of upper digestive and respiratory tract neoplasms [96, 97]. The inconsistent results between our study and prior meta-analyses could be explained by a prior study based on both prospective and retrospective observational studies, and the conclusion might be overestimated. Furthermore, the small number of studies in our study could explained an insignificant association of red and processed meat consumption with the risk of EC.

Our study indicated that meat intake was not associated with GC risk, and the conclusions were not affected by sensitivity and subgroup analyses. However, a prior meta-analysis suggested that red or processed meat consumption was associated with an increased risk of GC, whereas white meat could protect against GC risk [98]. Similar reasons to those for EC could explain these results. Moreover, DNA damage or oxidative stress caused by iron are important for the growth of *H. pylori*, which plays an important role in GC risk [99, 100]. Furthermore, the cooking method might play an important role in GC risk, including heterocyclic amines, polycyclic aromatic hydrocarbons, and salts [101, 102]. The conflicting results between the current study and prior studies could be explained by the fact that most of the included studies were case-control studies, and the causality relationship between meat consumption and GC was restricted.

Our study found that meat consumption plays an important role in the risk of CRC, CC, and RC, which is consistent with prior meta-analysis [11]. There were varies molecular pathways contributed the carcinogenesis across the regions of colon and rectum. The microsatellite instability, a CpG island methylator phenotype, and KRAS mutations were more evident for proximal colon cancers than rectal and distal colon tumors, whereas TP53 and APC mutations were more evident for rectal and distal colon tumors [103]. Moreover, heme iron could mediate the formation of intestinal carcinogenic compounds [95], and the progression of CRC could be affected by a specific bovine infectious factor [104]. Furthermore, chemical carcinogens, including heterocyclic amines and polycyclic aromatic hydrocarbons, play an important role in the risk of CRC. In addition, subgroup analyses revealed that the most significant associations were observed in Western countries, longer follow-up duration, and studies with moderately adjusted levels, which could be explained by the dietary structure between Western and Eastern countries; longer follow-up could result in a greater number of new cancers, and the power was stronger; and only a smaller number of included studies reported studies with moderately adjusted levels, and the pooled conclusion was not stable.

No significant association between meat consumption and PC risk was observed, which was not consistent with a previous meta-analysis that suggested that processed meat consumption was associated with an increased risk of PC, and red meat consumption was associated with an increased risk of PC in men but not in women [105]. They explained these results through *N*-nitroso compounds that could reach the pancreas via the bloodstream and act as potential carcinogens [106]. Subgroup analyses indicated that total red and processed meat consumption were associated with an increased risk of PC if pooled studies were conducted in Eastern countries, which could be explained by cooking methods for red meat and a smaller number of studies in this subgroup.

Higher meat consumption was not associated with the risk of HCC, irrespective of whether it is red, processed, or total red and processed meat, which is consistent with prior meta-analysis [107]. However, the association of red meat consumption with the risk of HCC was not stability, and red meat consumption was associated with an increased risk of HCC. The potential mechanism could be high levels of cholesterol and saturated fat in red meat is significantly related to the progression of cancer. Moreover, subgroup analyses revealed that processed meat was associated with a reduced risk of HCC when the follow-up duration was < 10.0 years. These results could be explained by the fact that HCC progression is significantly related to socioeconomic status, which could affect meat consumption [108, 109]. Other influencing factors included selection bias, random errors, and various adjusted levels.

Some limitations of this study should be acknowledged. First, the meat consumption definition was assessed using various methods, which could affect the actual acceptable daily consumption and the effect estimates for gastrointestinal cancer; (2) the heterogeneity across included studies was not fully explained by using sensitivity and subgroup analyses; (3) adjusted factors among included studies were different, which could affect the effect estimate for the relationship between meat consumption and gastrointestinal cancer risk; (4) the ratio between subgroups was calculated based on indirect comparisons, and the results needed further direct comparison; and (5) the analysis based on pooled data and individual data were not available, which restricted detailed analyses.

## Conclusions

Our study found that higher meat consumption was associated with an increased risk of CRC, CC, and RC irrespective of whether it was red, processed, or total red and processed meat that was consumed. Moreover, the strength of the relationship between meat consumption and gastrointestinal cancer risk could be affected by follow-up duration and adjusted level. Further large-scale prospective studies should be performed to assess the potential effects of dietary interventions on the risk of gastrointestinal cancers.

### Supplementary Information

**Additional file 1**

**Additional file 2**

## Data Availability

The datasets used and/or analysed during the current study are available from the corresponding author on reasonable request.

## Abbreviations

IARC: International Agency for Research on Cancer
CRC: Colorectal cancer
PC: Pancreatic cancer
EC: Esophageal cancer
GC: Gastric cancer
CC: Colon cancer
RC: Rectal cancer
HCC: Hepatocellular carcinoma
NOS: Newcastle-Ottawa Scale
RR: Relative risk
CI: Confidence interval
