# Supplementary material for: Association of meat consumption with the risk of gastrointestinal cancers: a systematic review and meta-analysis
Source: BMC Cancer. 2023 Aug 23;23:782. doi: 10.1186/s12885-023-11218-1 (PMC10463360; doi:10.1186/s12885-023-11218-1)
Supplement: Supplementary file 1 — Additional file 1 [file 12885_2023_11218_MOESM1_ESM.docx]

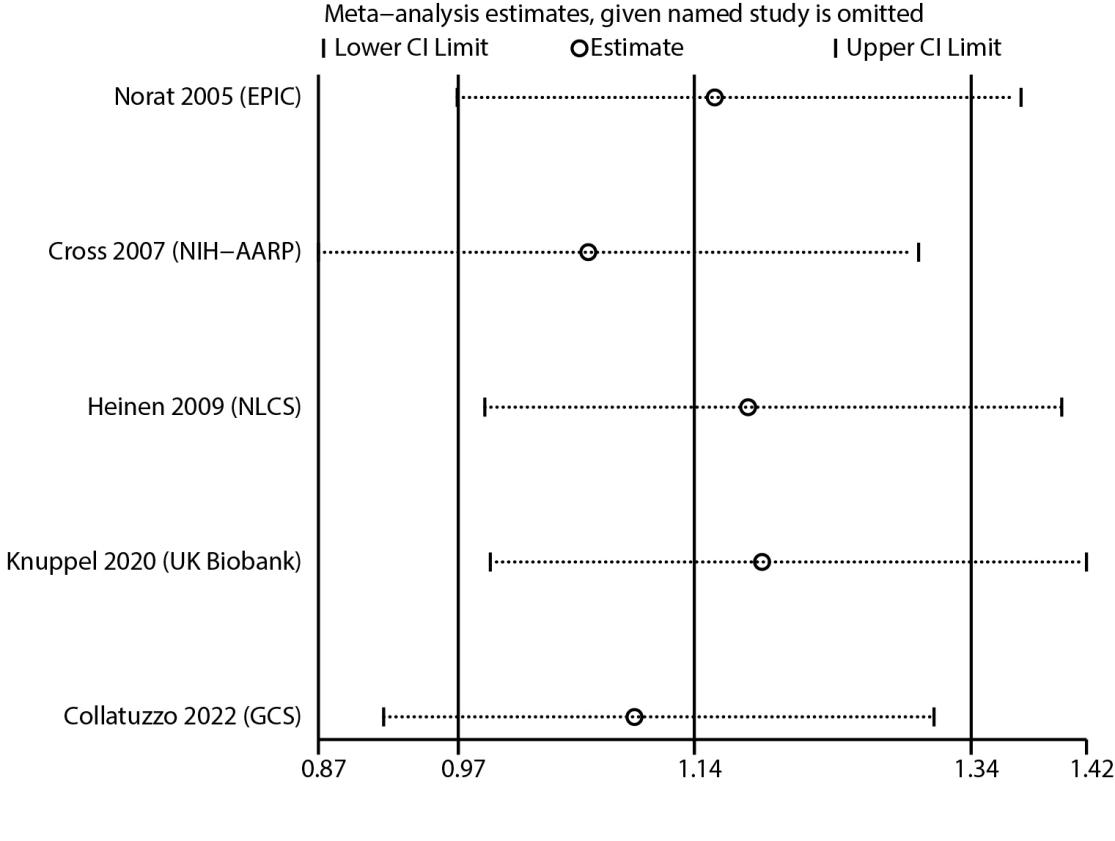


Figure S1. Sensitivity analysis for the relation between red meat intake andEC risk


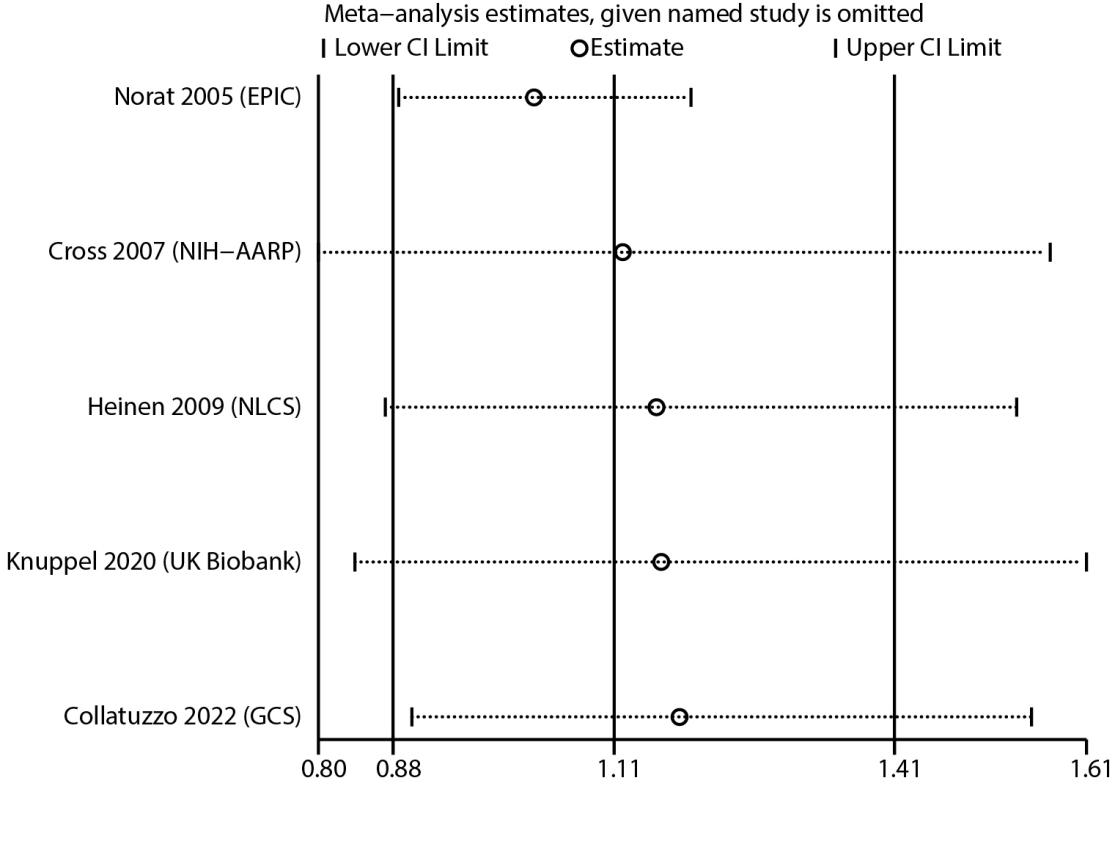


Figure S2. Sensitivity analysis for the relation between proceed meat intake andEC risk


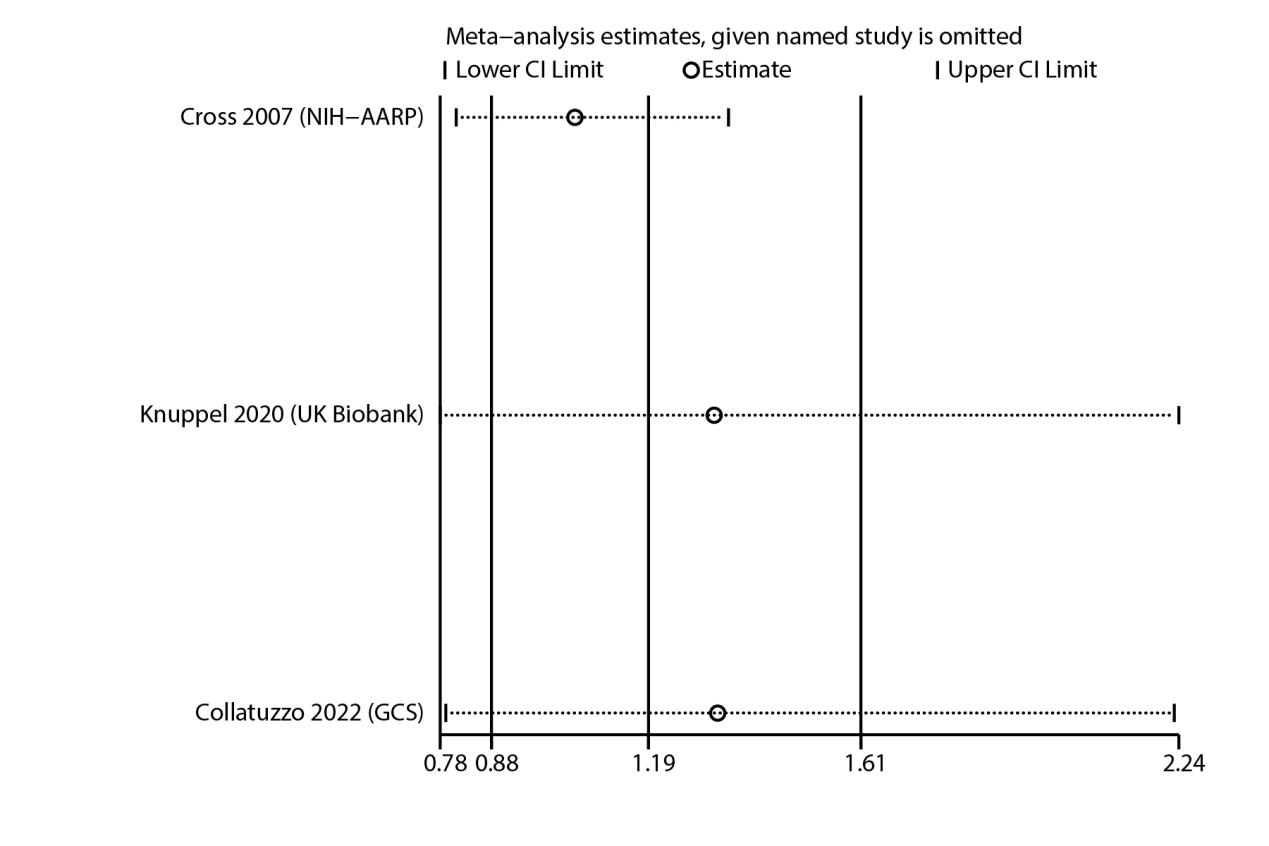


Figure S3. Sensitivity analysis for the relation betweentotal red and processed meat intake andEC risk


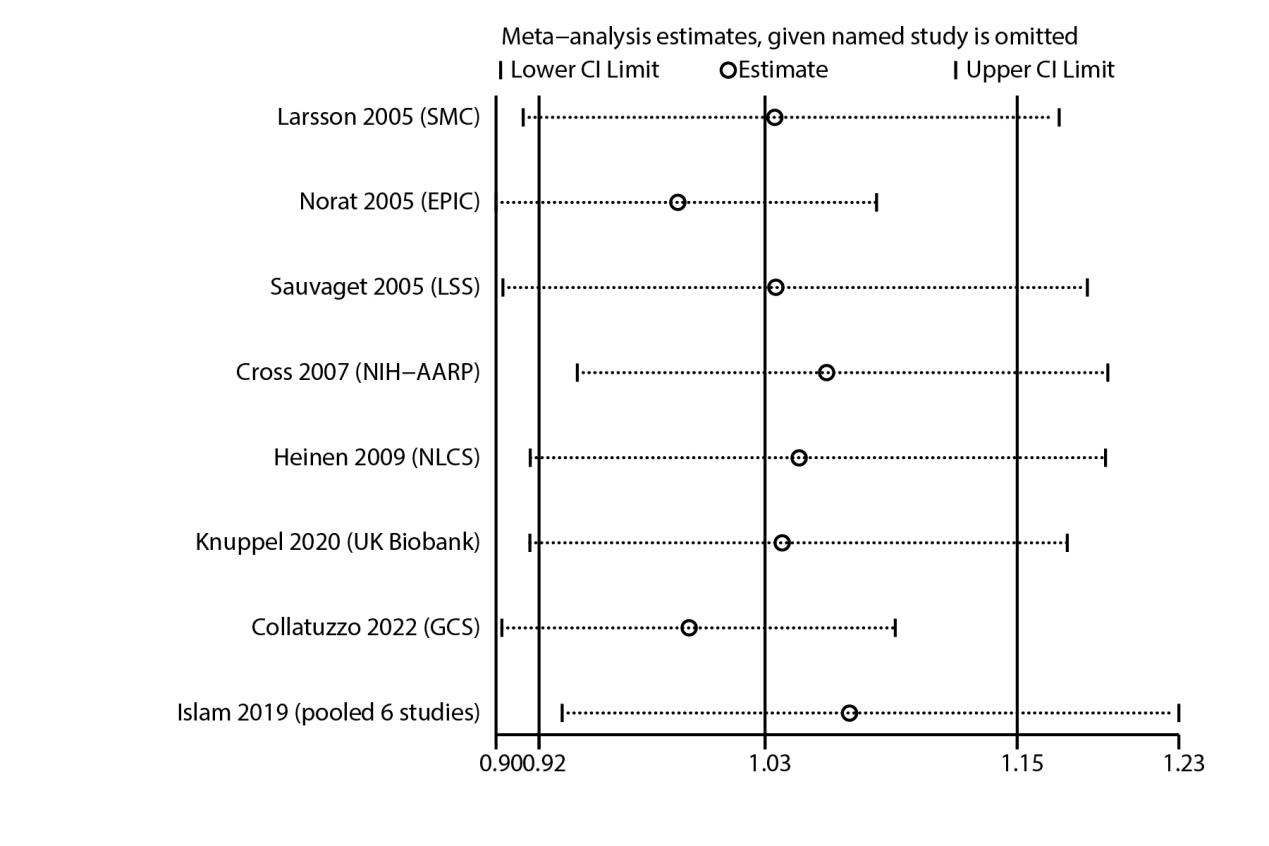


Figure S4. Sensitivity analysis for the relation between red meat intake and GC risk


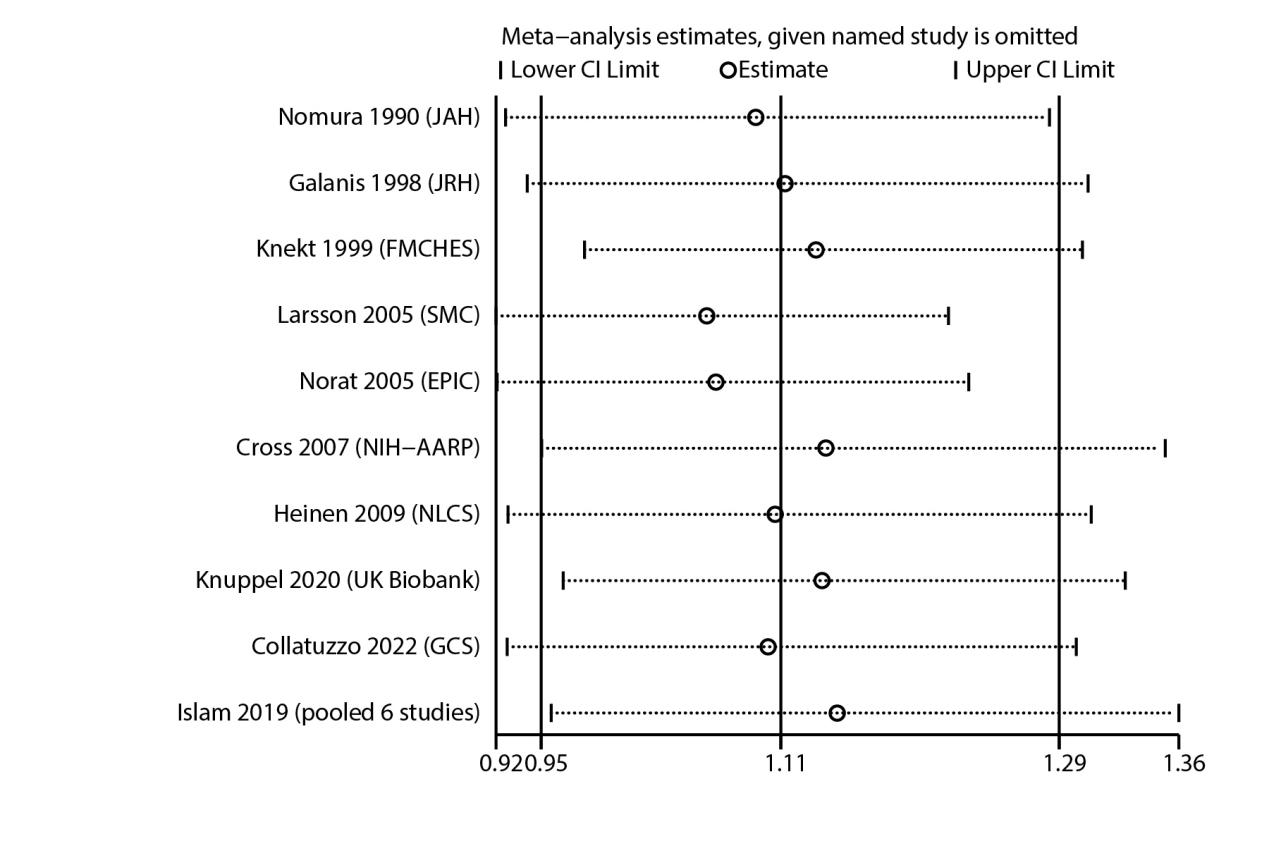


Figure S5. Sensitivity analysis for the relation between proceed meat intake and GC risk


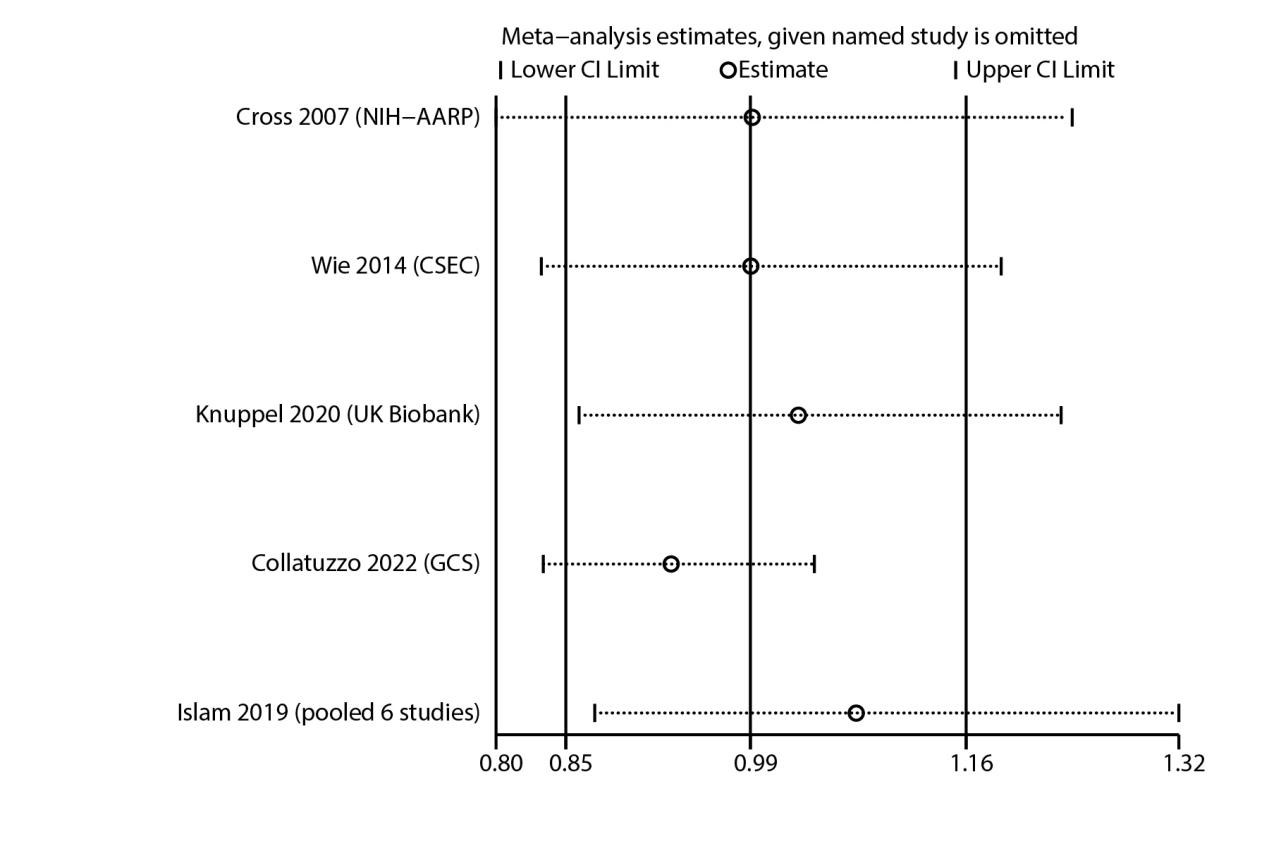


Figure S6. Sensitivity analysis for the relation betweentotal red and processed meat intake and GC risk


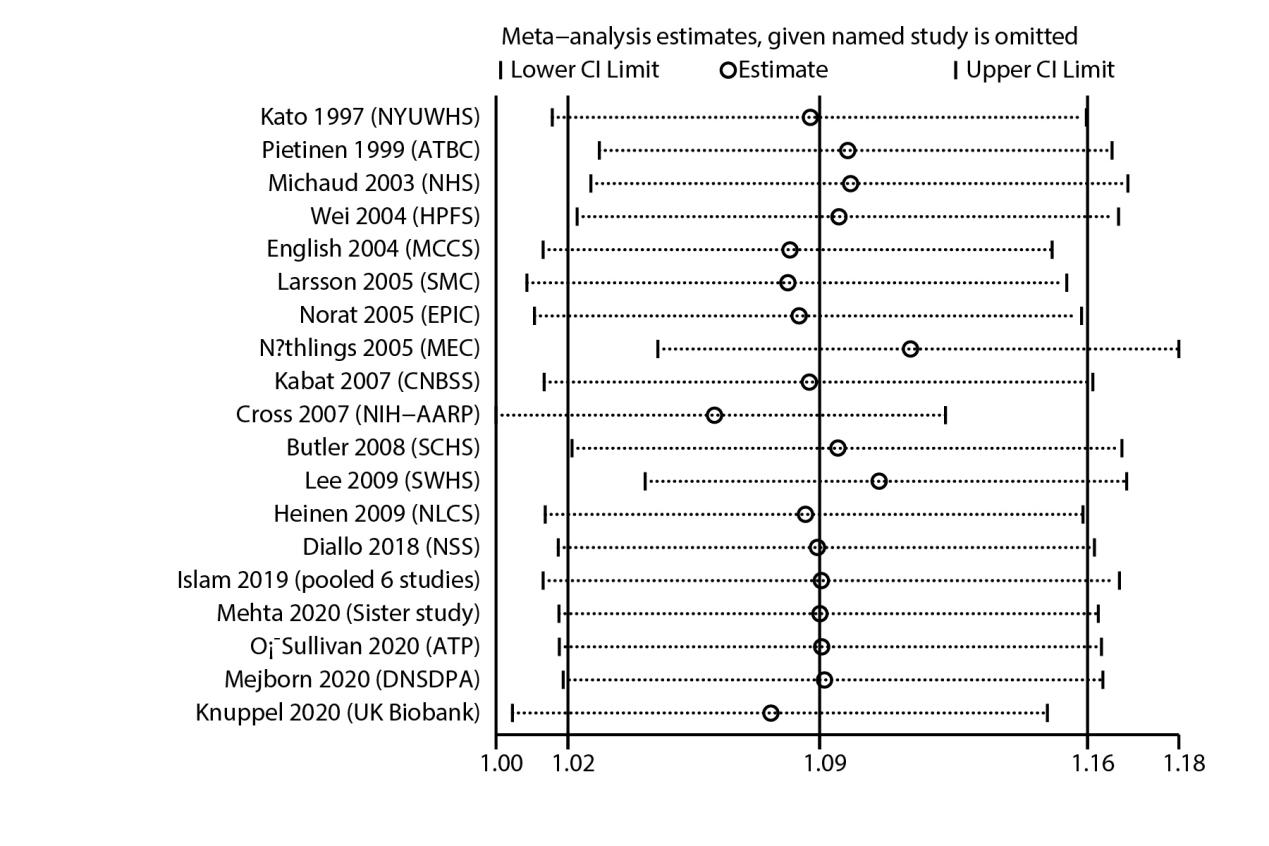


Figure S7. Sensitivity analysis for the relation between red meat intake and CRC risk


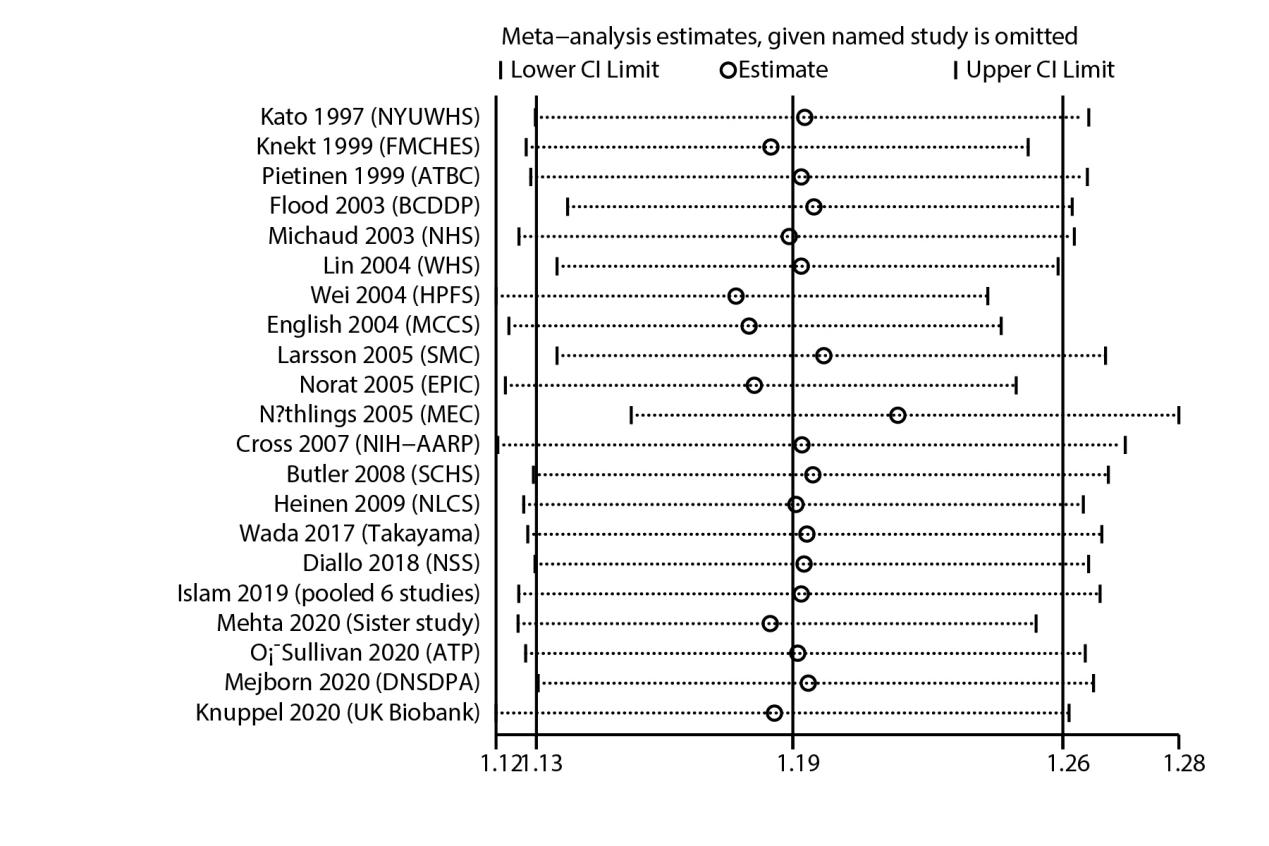


Figure S8. Sensitivity analysis for the relation between proceed meat intake and CRC risk


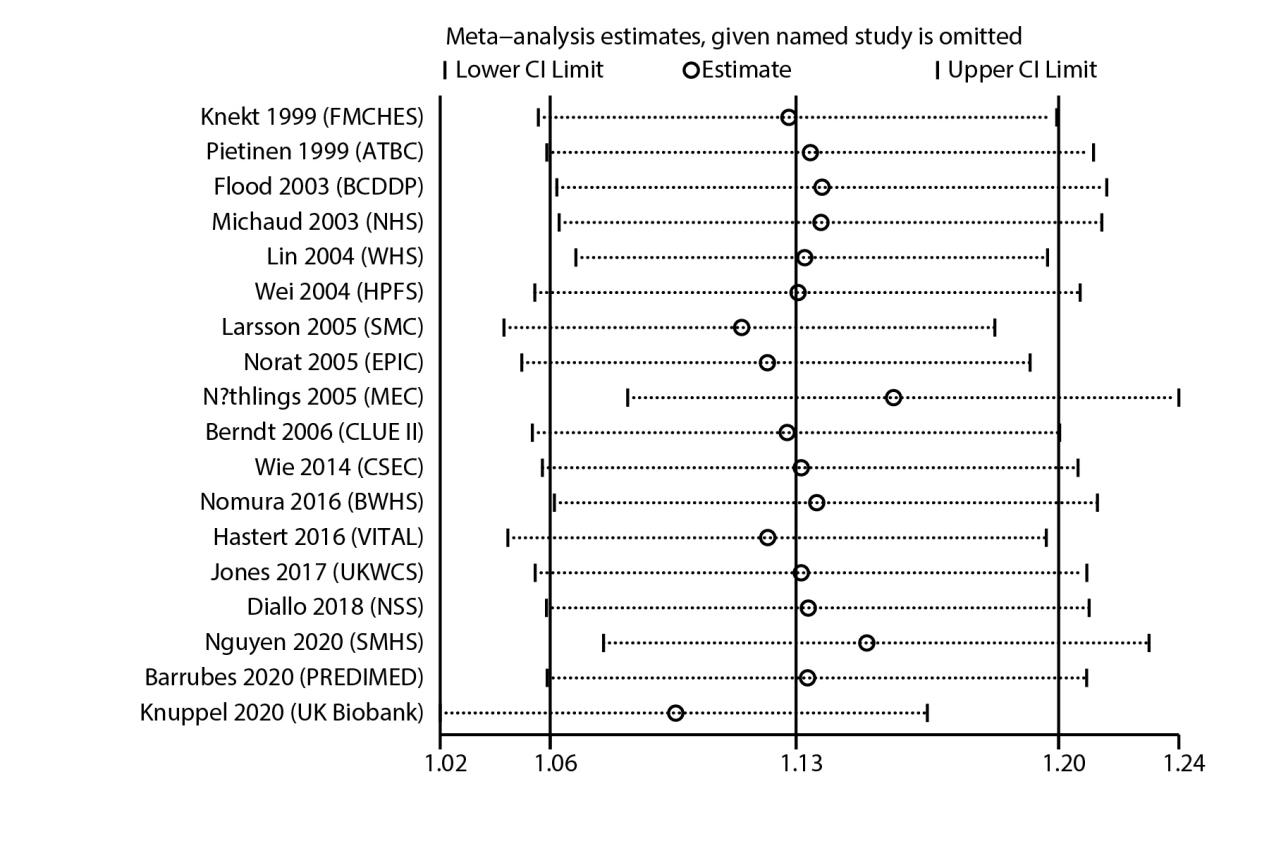


Figure S9. Sensitivity analysis for the relation betweentotal red and processed meat intake and CRC risk


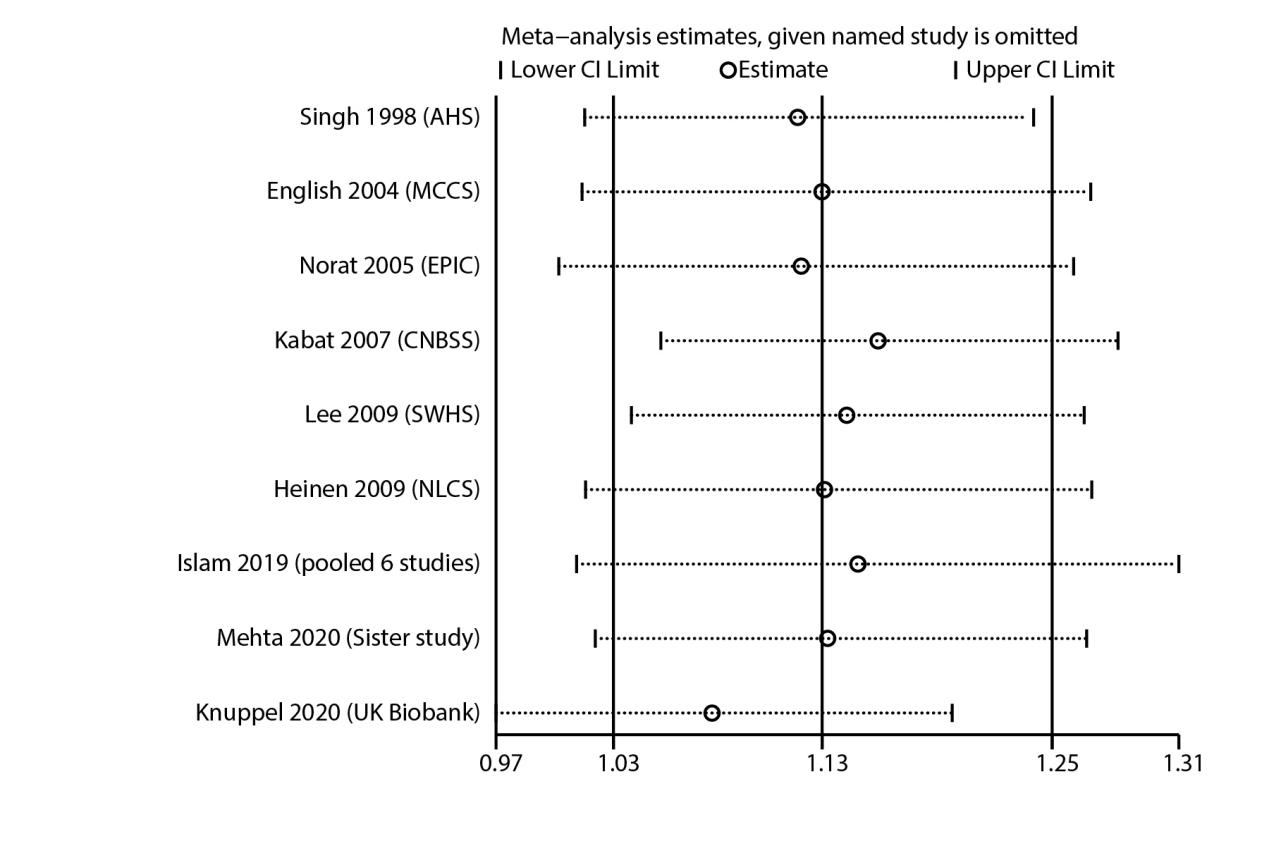


Figure S10. Sensitivity analysis for the relation between red meat intake and CC risk


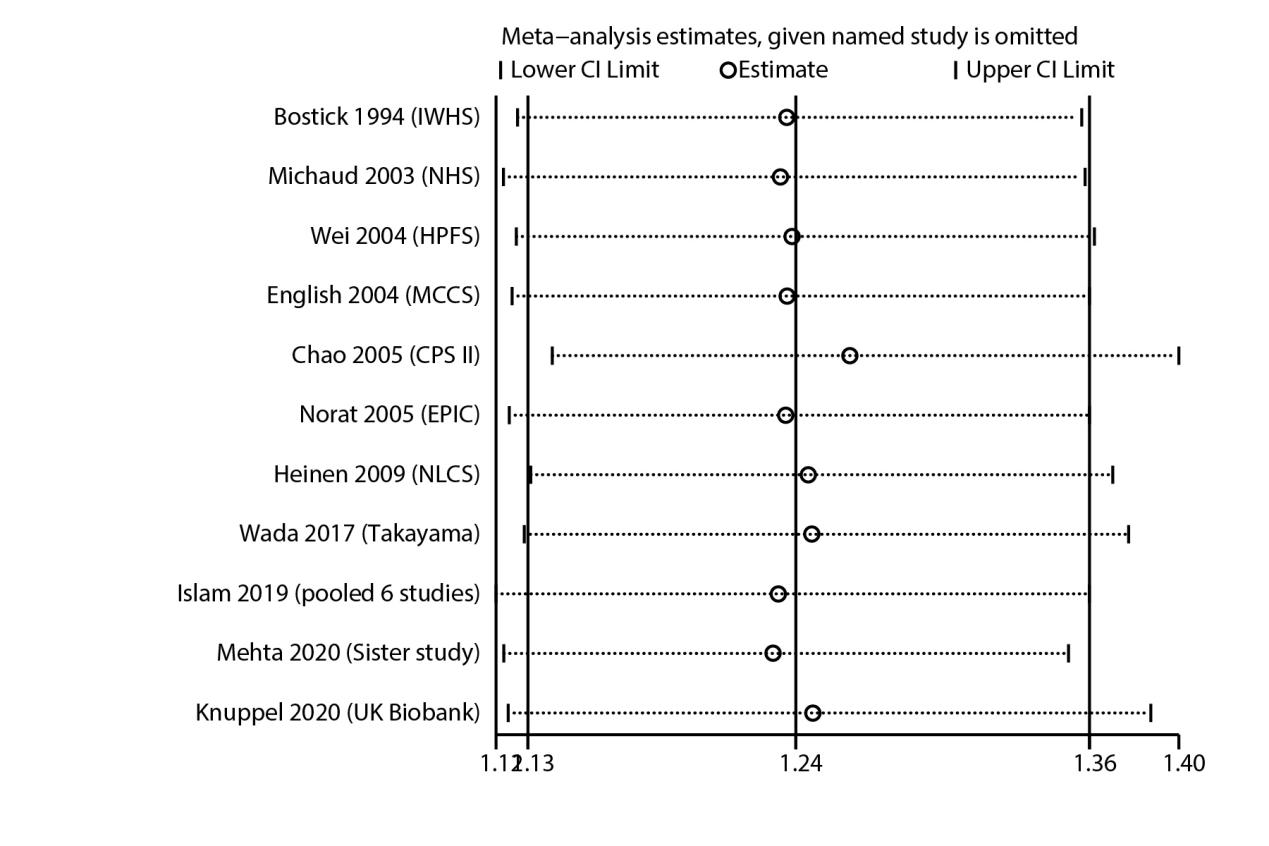


Figure S11. Sensitivity analysis for the relation between proceed meat intake and CC risk


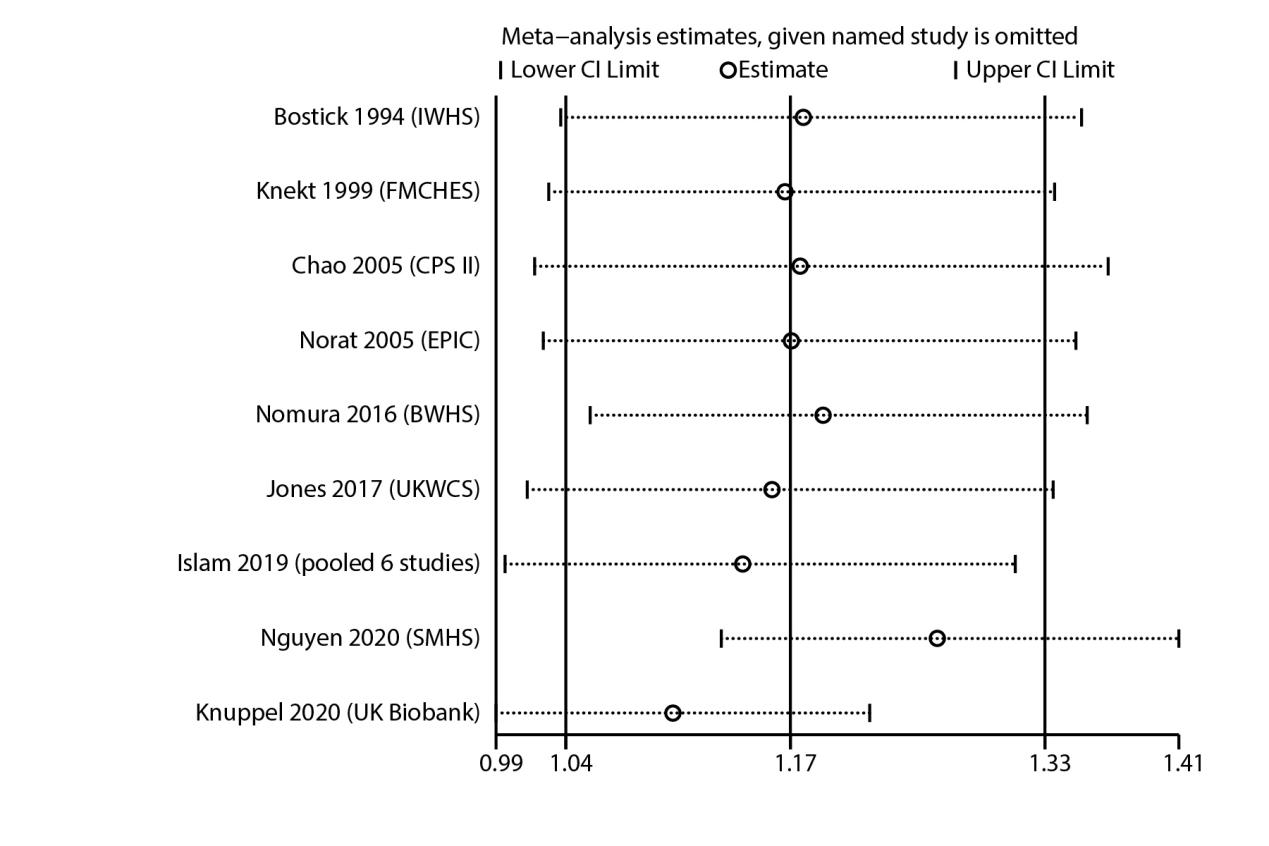


Figure S12. Sensitivity analysis for the relation betweentotal red and processed meat intake and CC risk


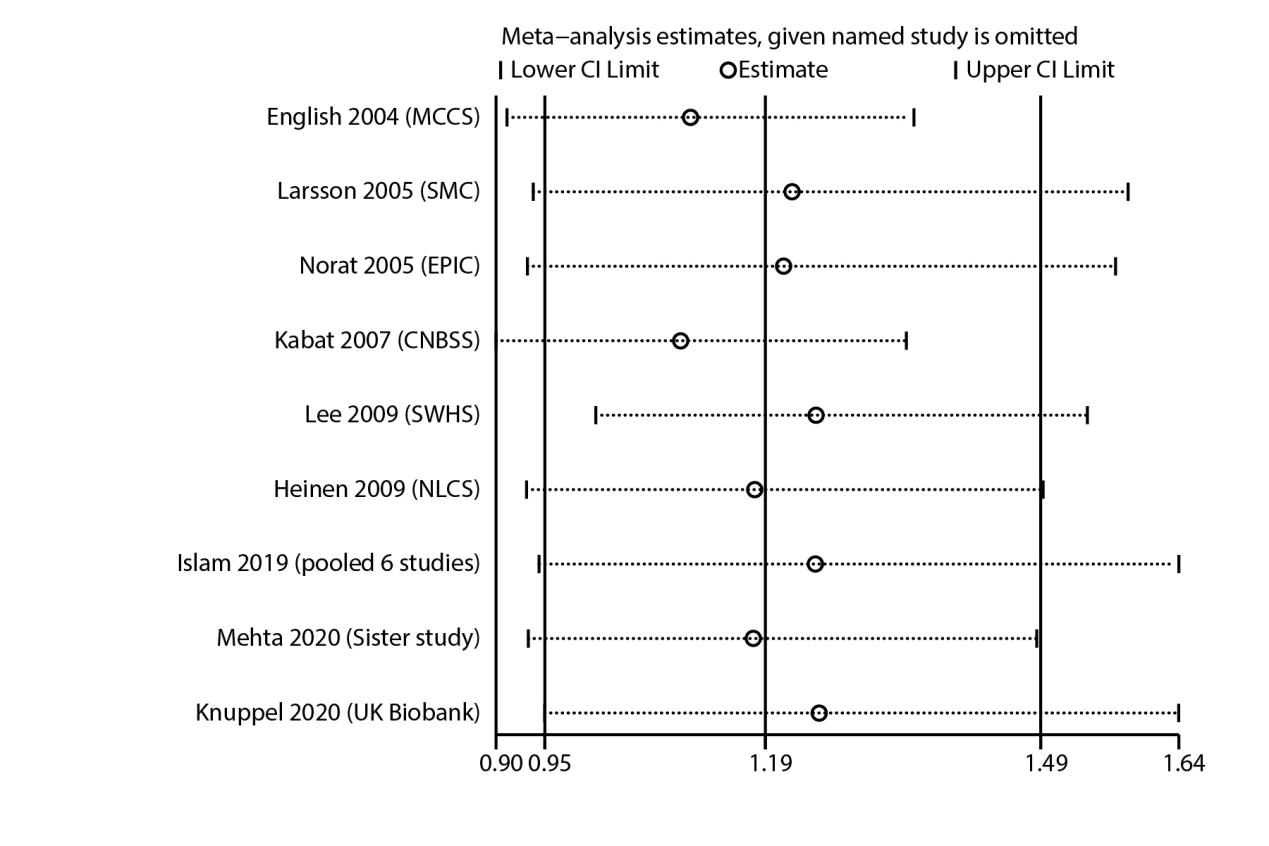


Figure S13. Sensitivity analysis for the relation between red meat intake and RC risk


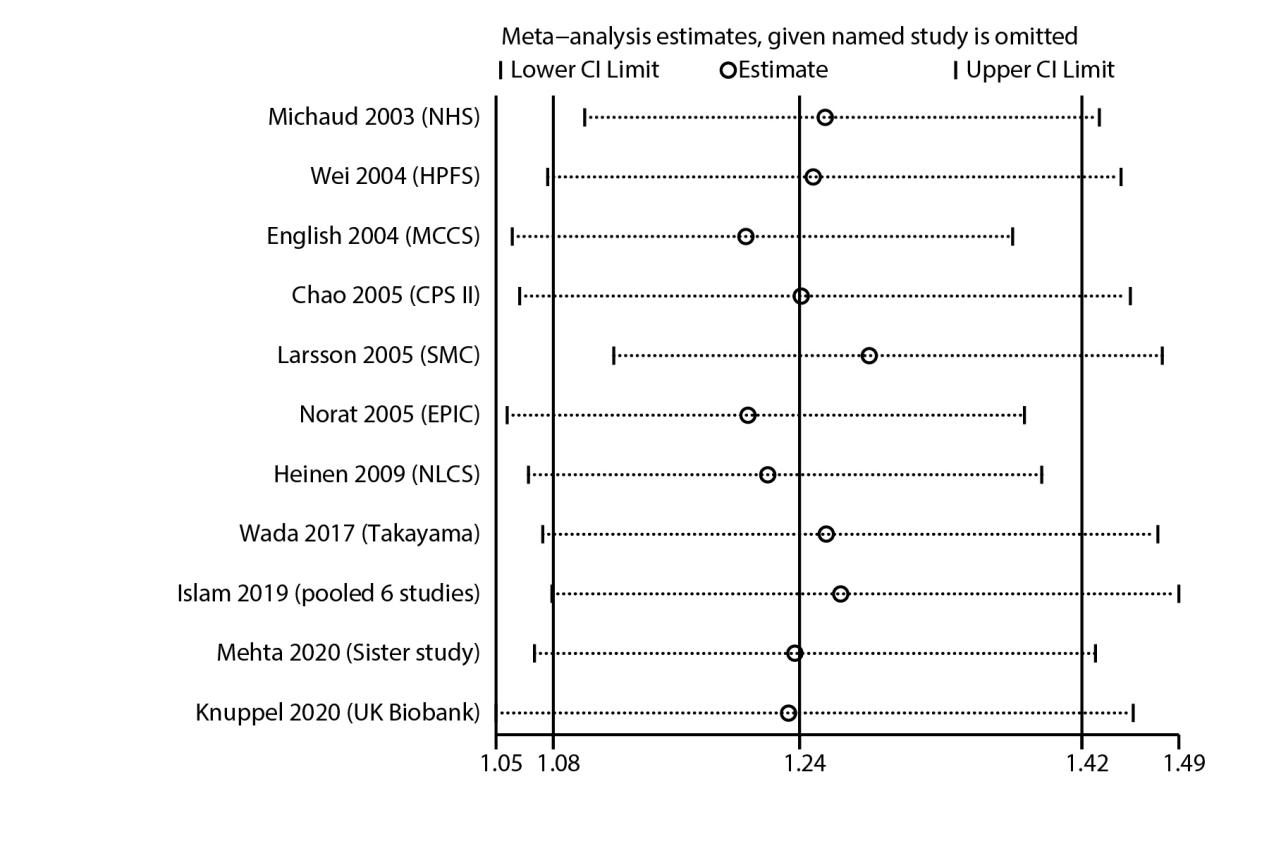


Figure S14. Sensitivity analysis for the relation between proceed meat intake and RC risk


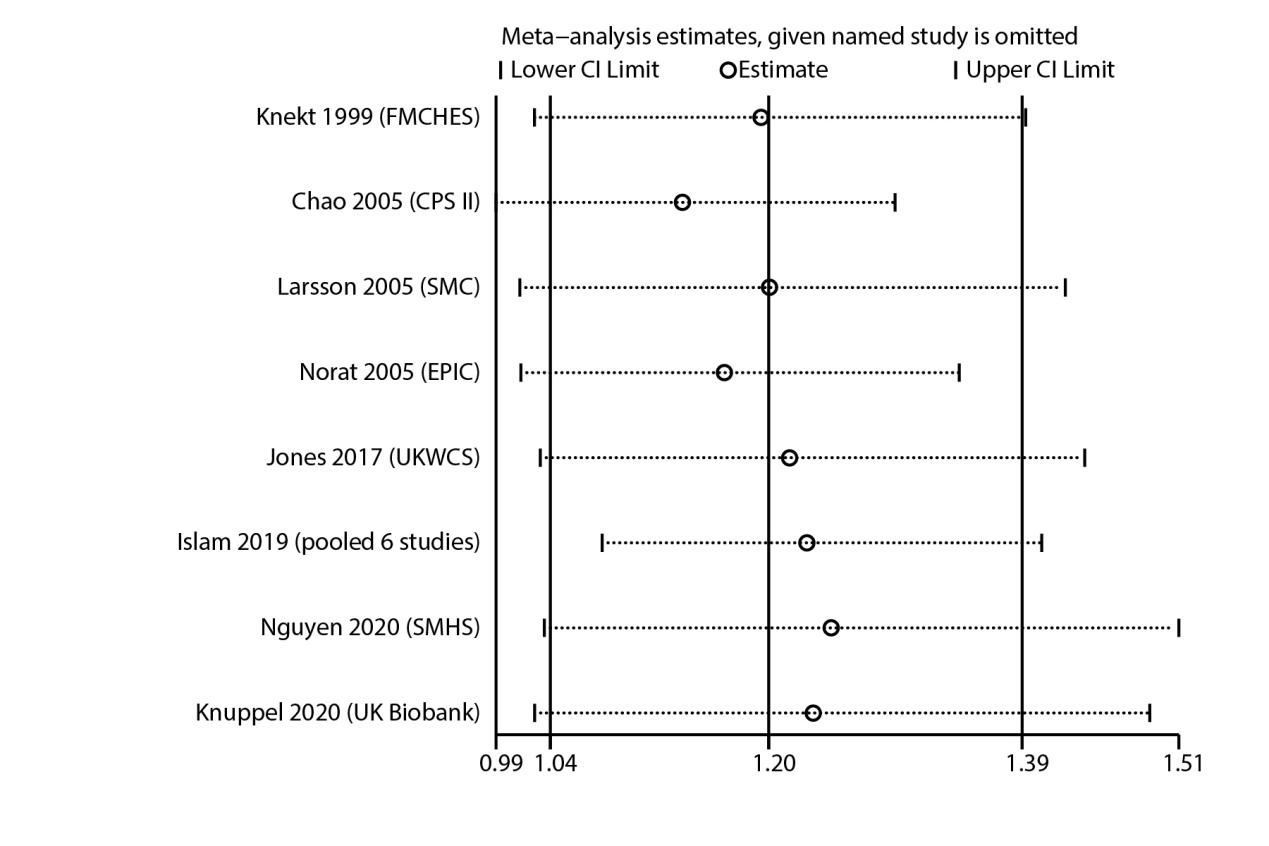


Figure S15. Sensitivity analysis for the relation betweentotal red and processed meat intake and RC risk


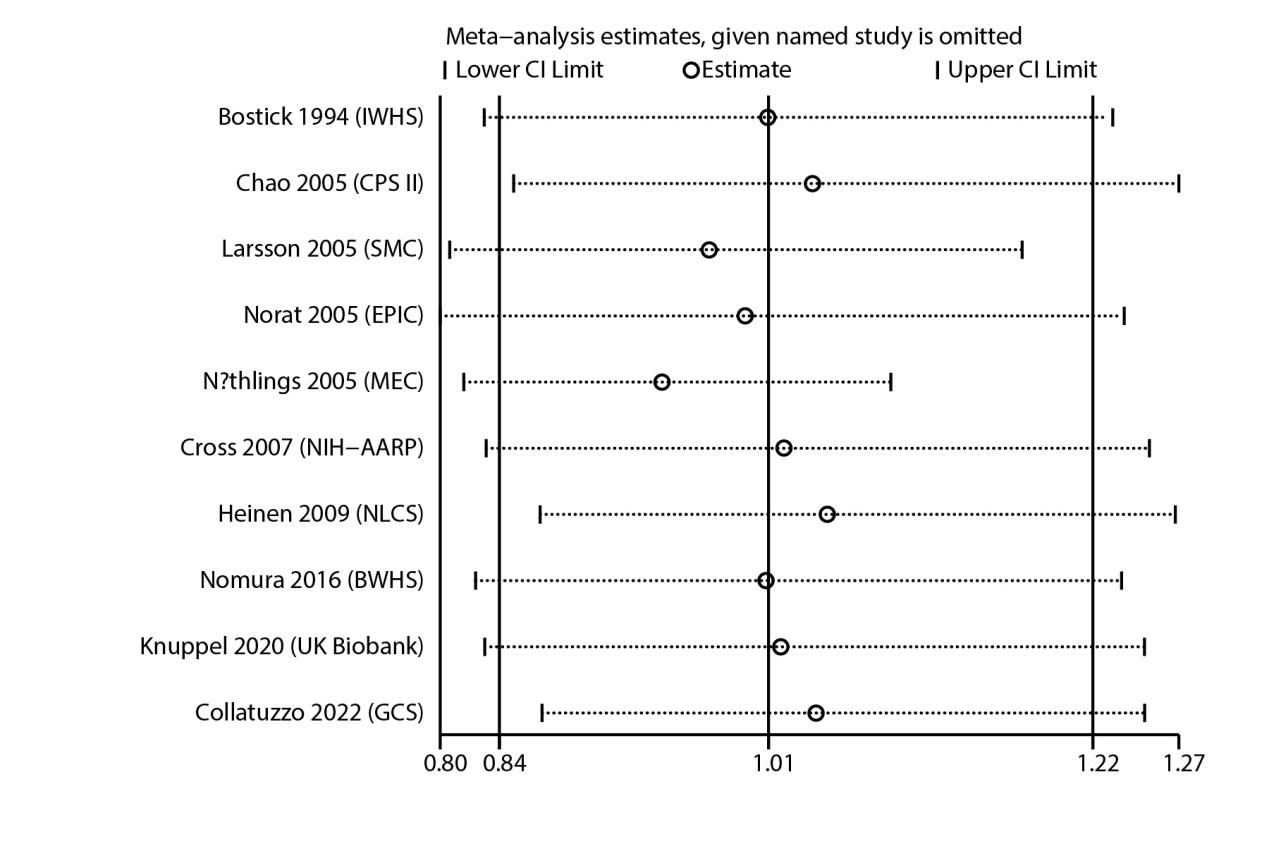


Figure S16. Sensitivity analysis for the relation between red meat intake and PC risk


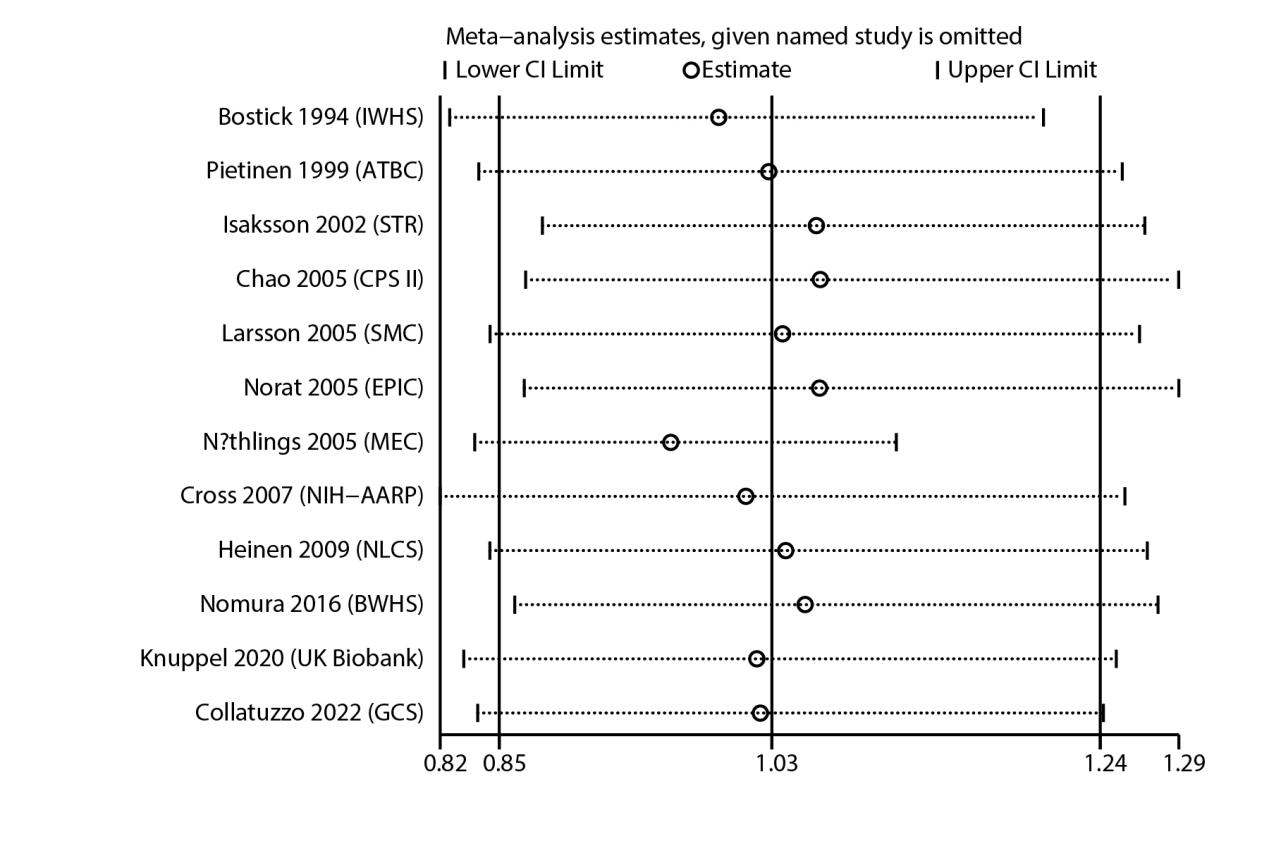


Figure S17. Sensitivity analysis for the relation between proceed meat intake and PC risk


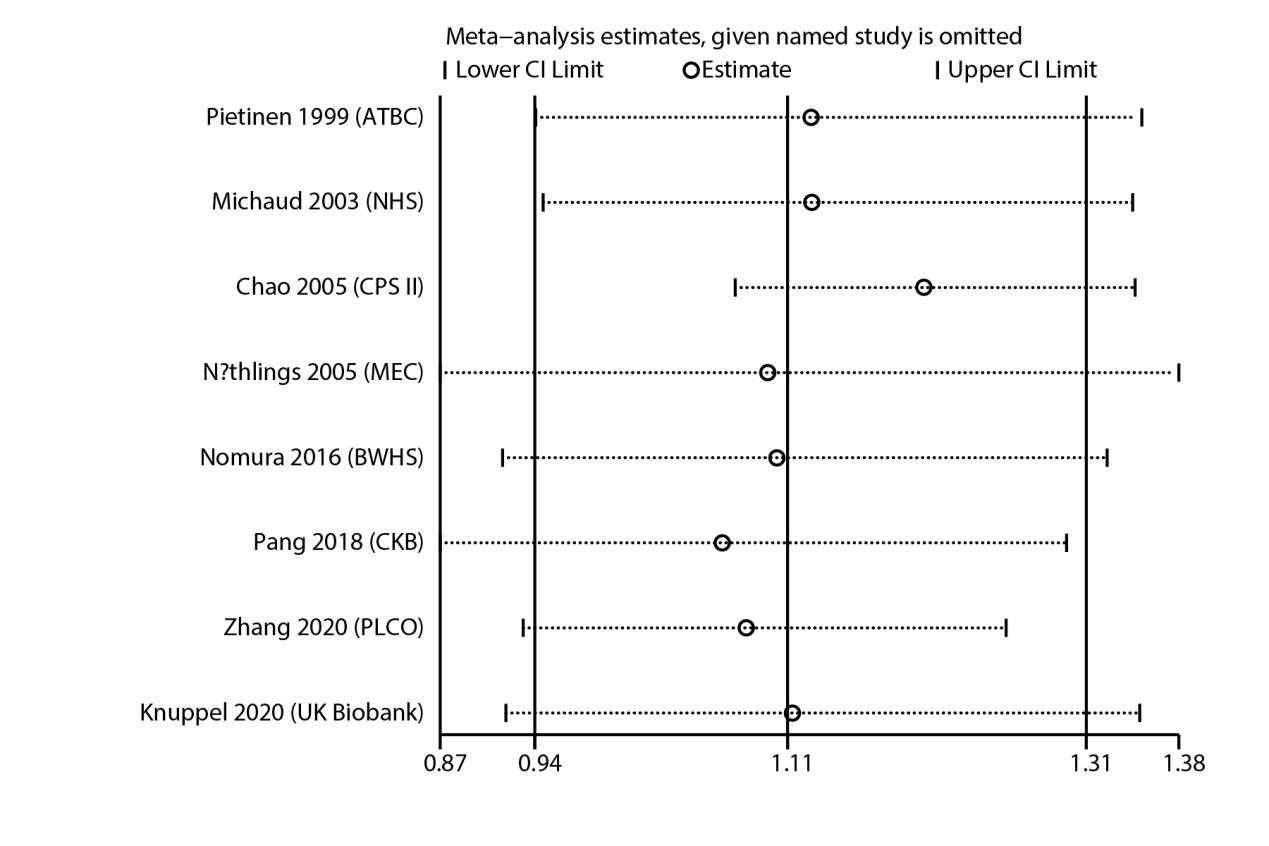


Figure S18. Sensitivity analysis for the relation betweentotal red and processed meat intake and PC risk


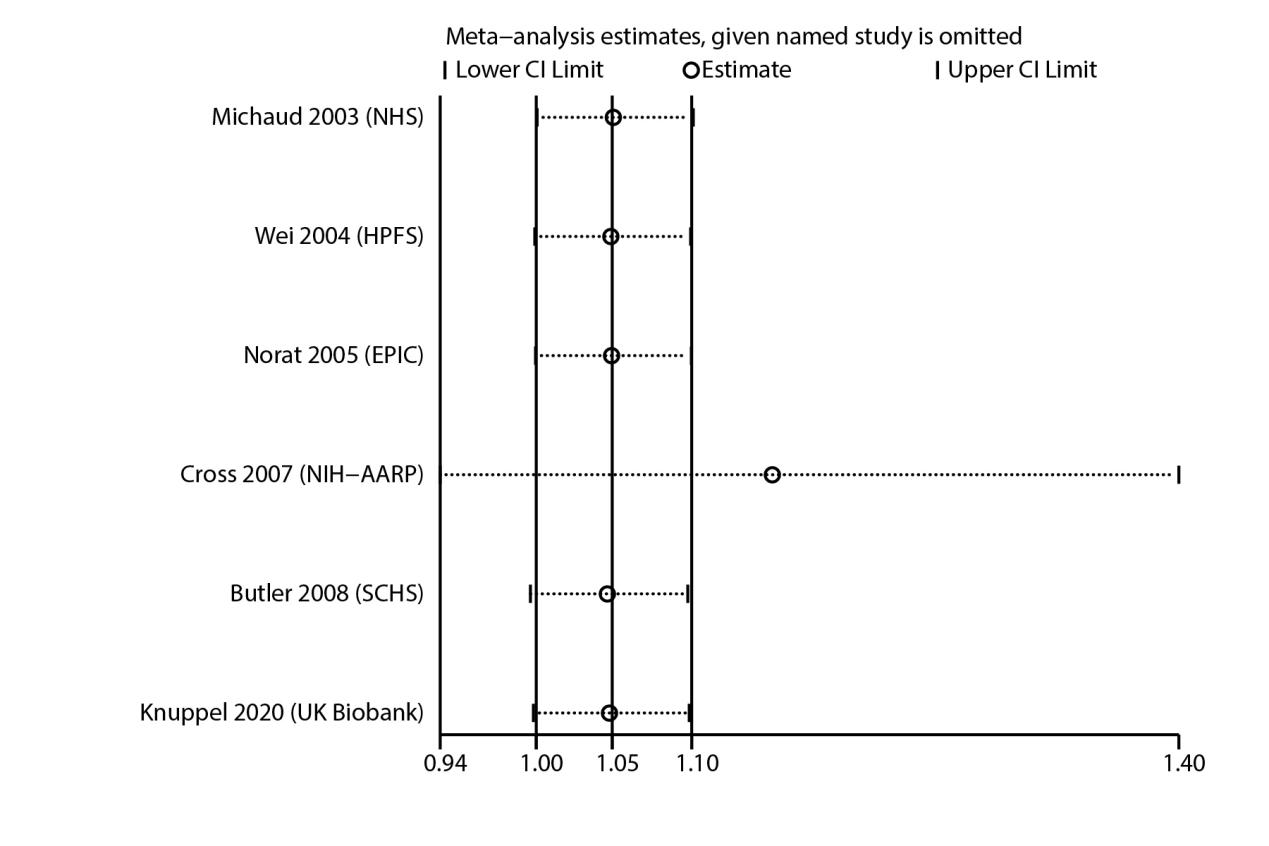


Figure S19. Sensitivity analysis for the relation between red meat intake and HCC risk


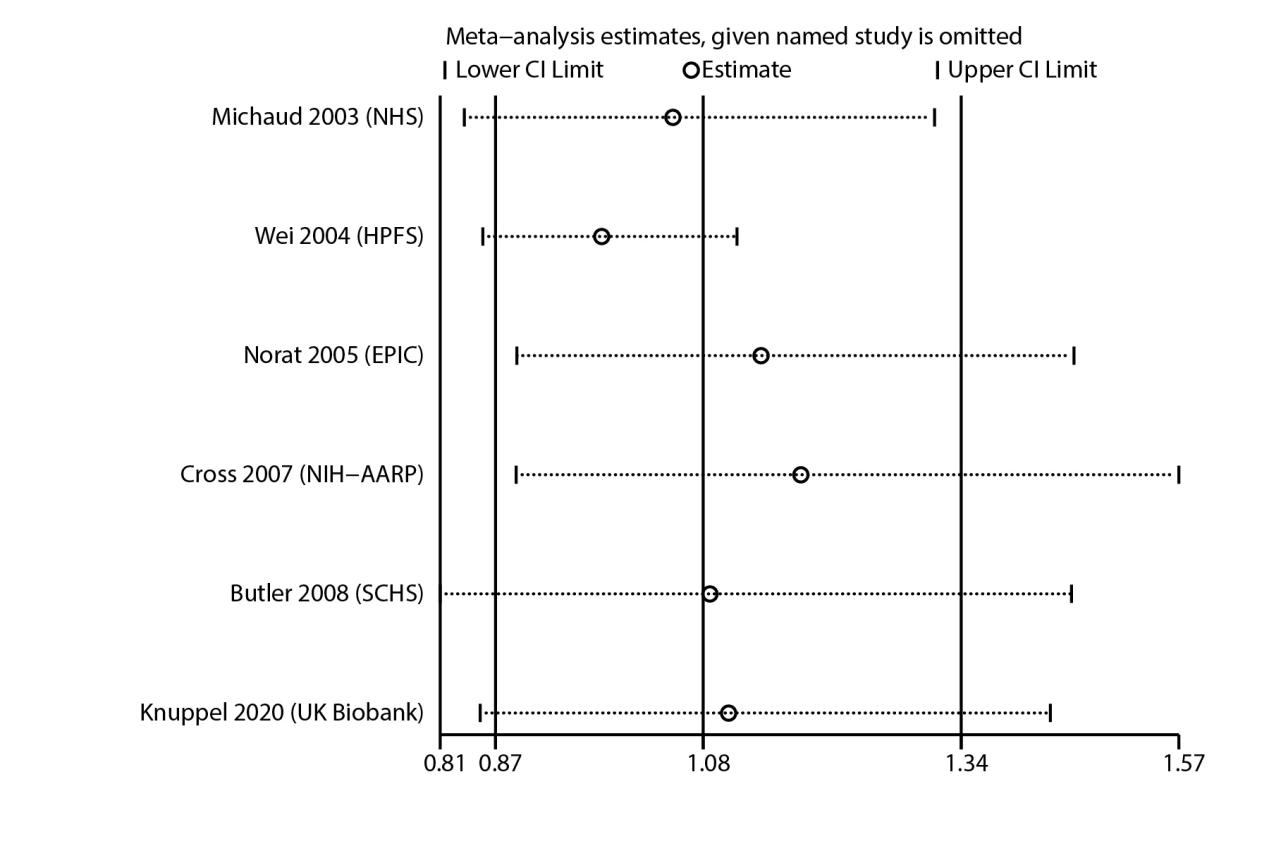


Figure S20. Sensitivity analysis for the relation between proceed meat intake and HCC risk


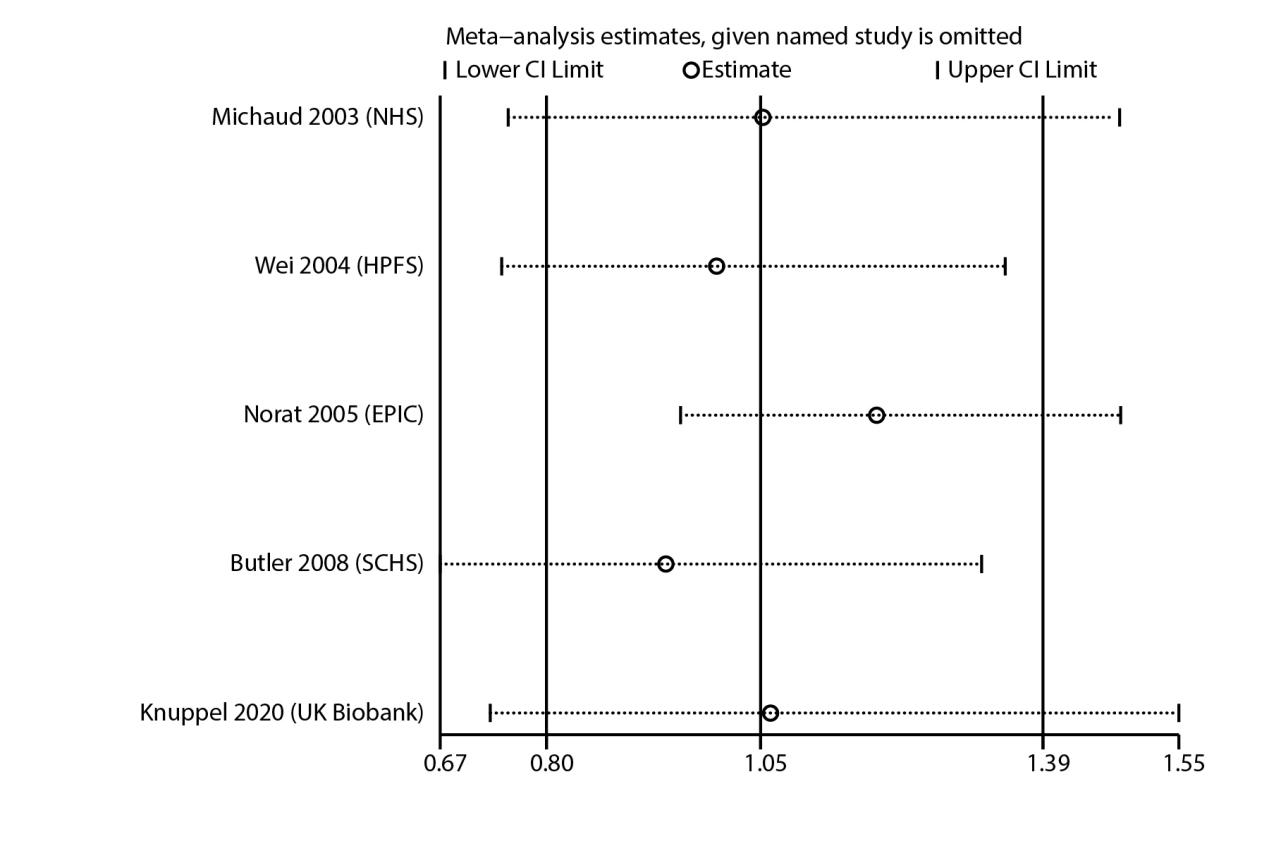


Figure S21. Sensitivity analysis for the relation betweentotal red and processed meat intake and HCC risk
