# Supplementary material for: Association of meat consumption with the risk of gastrointestinal cancers: a systematic review and meta-analysis
Source: BMC Cancer. 2023 Aug 23;23:782. doi: 10.1186/s12885-023-11218-1 (PMC10463360; doi:10.1186/s12885-023-11218-1)
Supplement: Supplementary file 2 — Additional file 2 [file 12885_2023_11218_MOESM2_ESM.docx]

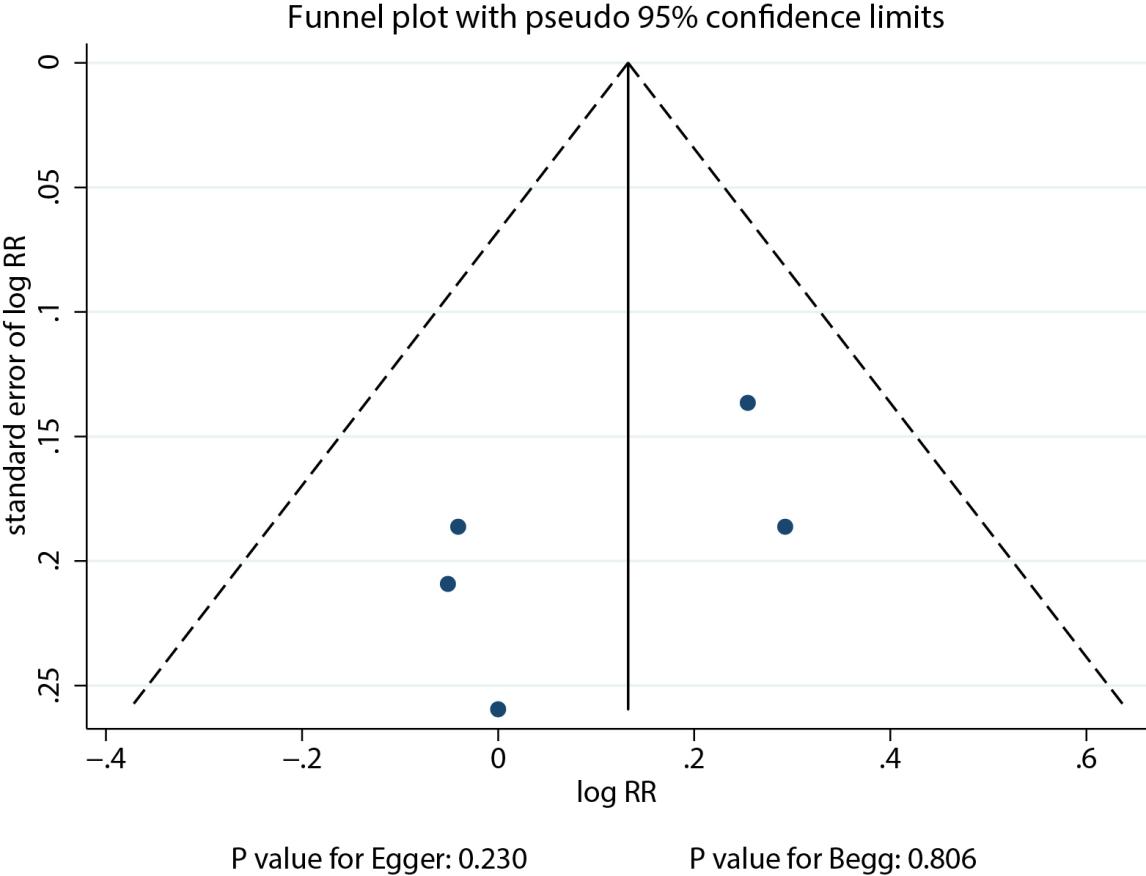


Figure S1. Funnel plot for the relation between red meat intake and EC risk


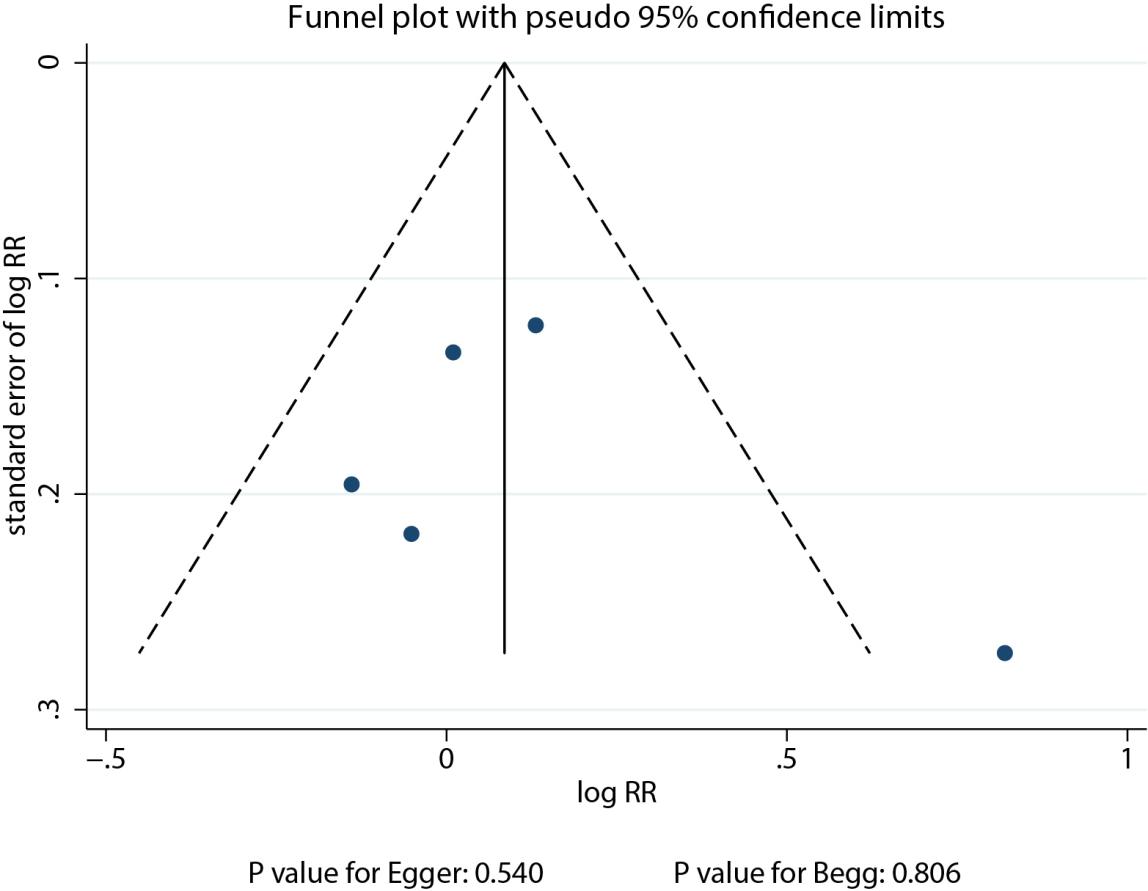


Figure S2. Funnel plot for the relation between proceed meat intake and EC risk


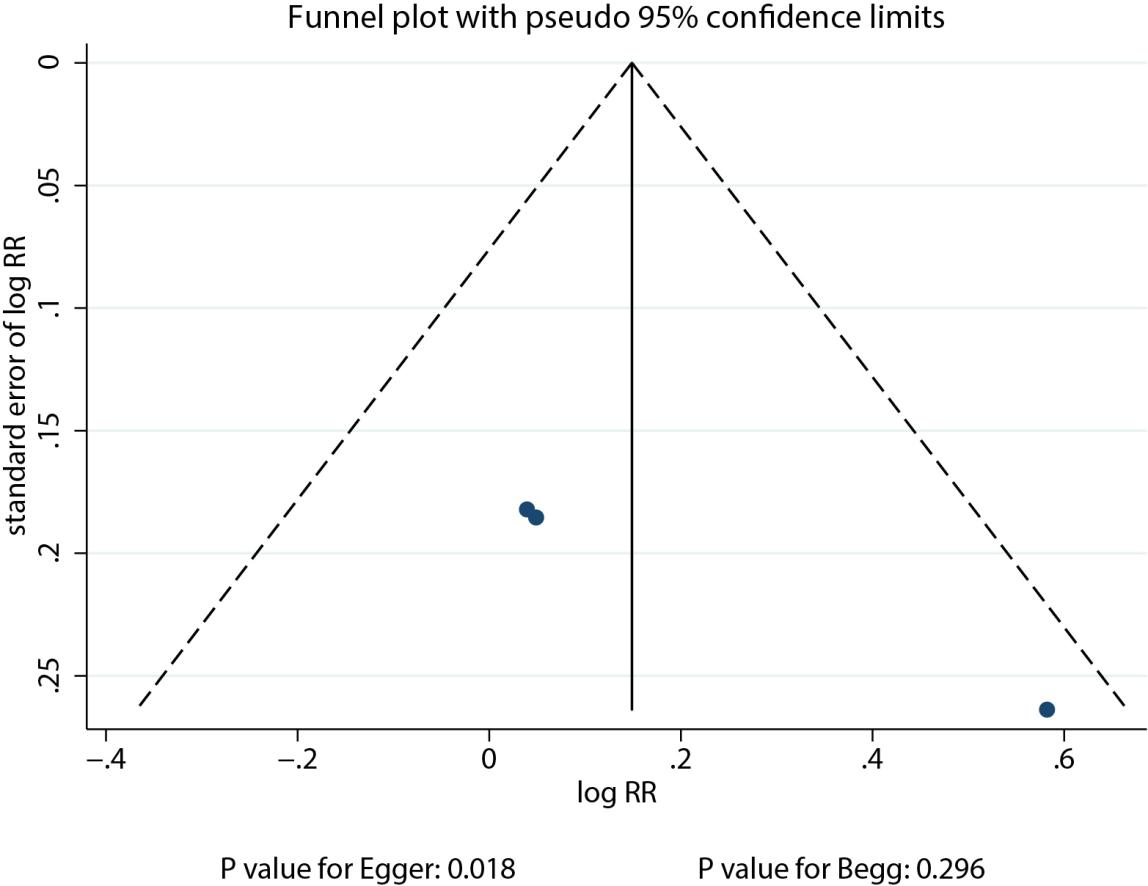


Figure S3. Funnel plot for the relation between total red and processed meat intake and EC risk


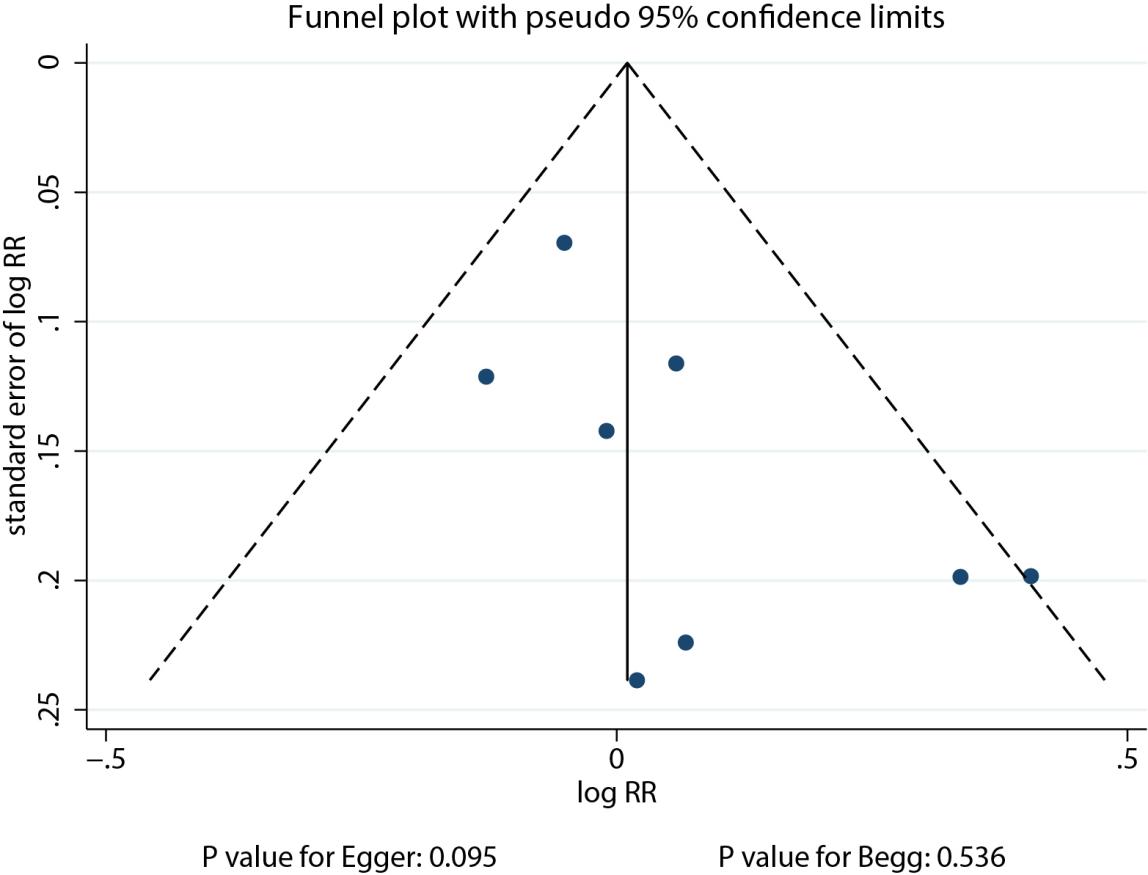


Figure S4. Funnel plot for the relation between red meat intake and GC risk


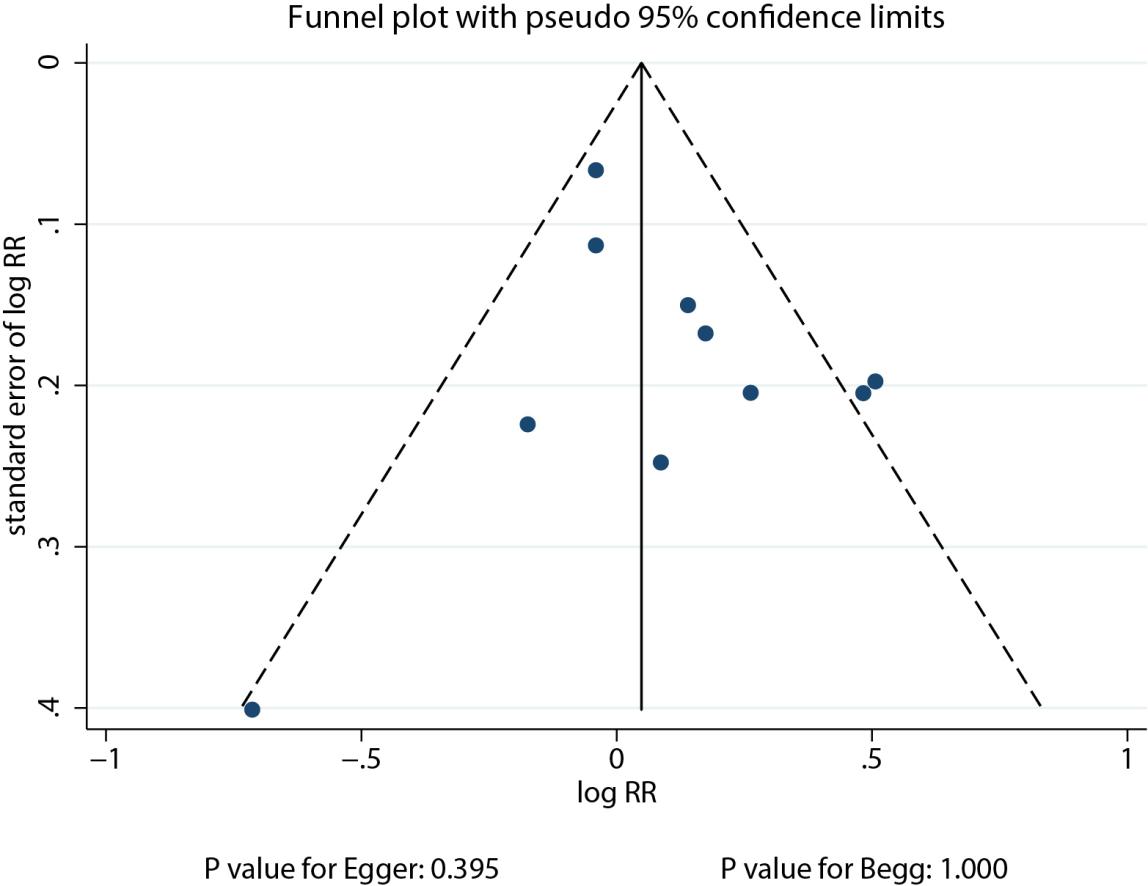


Figure S5. Funnel plot for the relation between proceed meat intake and GC risk


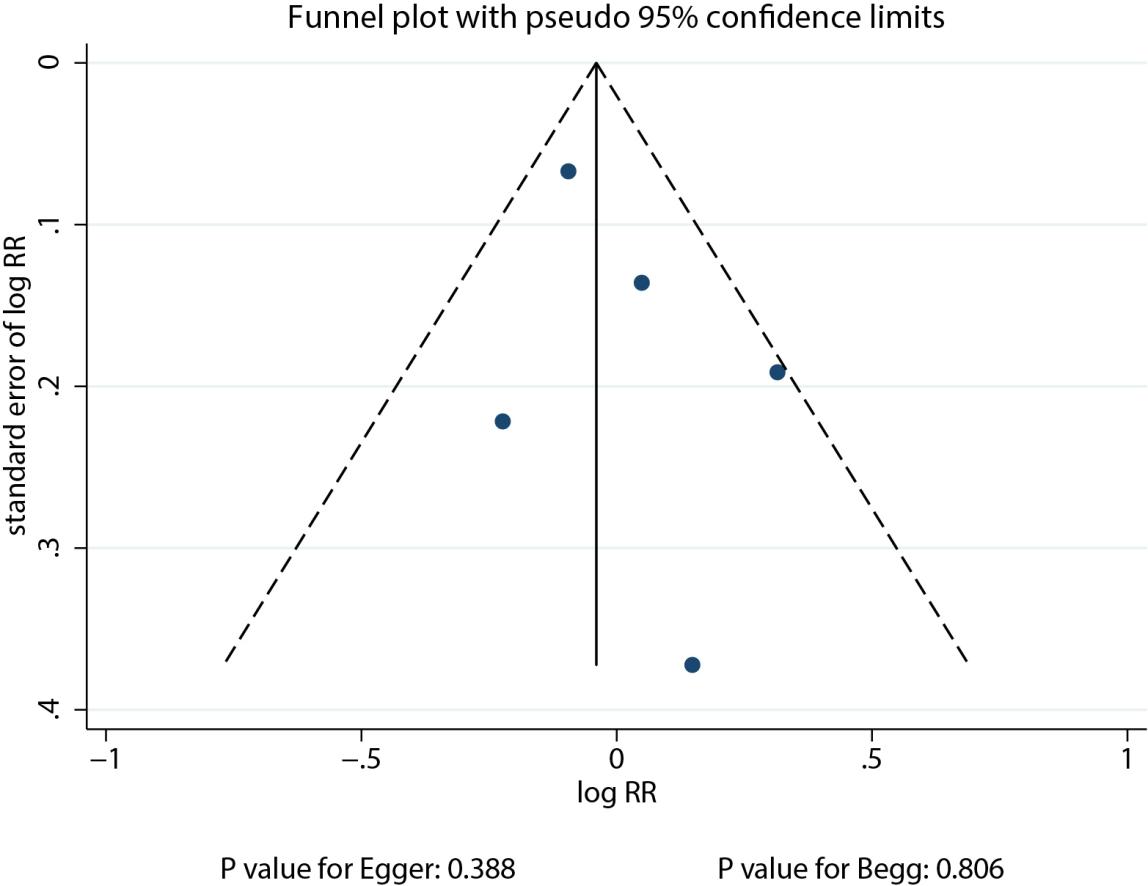


Figure S6. Funnel plot for the relation between total red and processed meat intake and GC risk


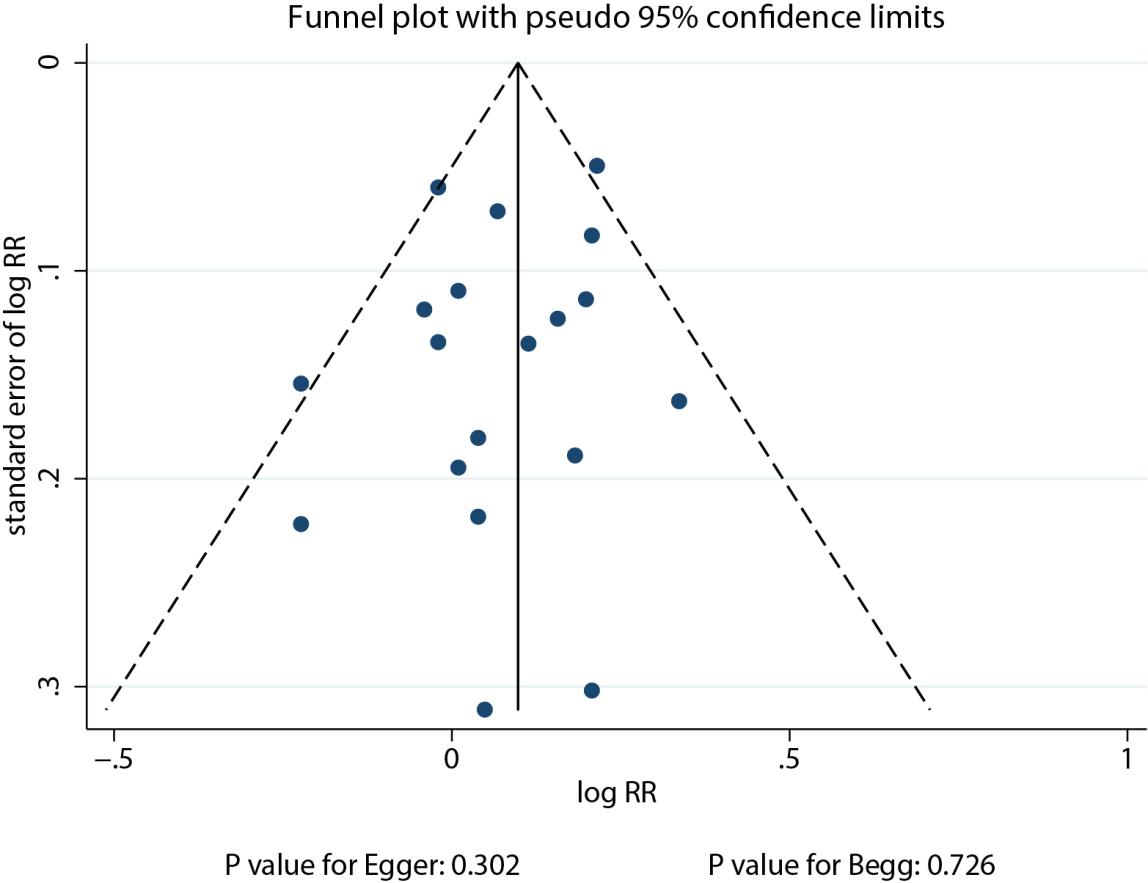


Figure S7. Funnel plot for the relation between red meat intake and CRC risk


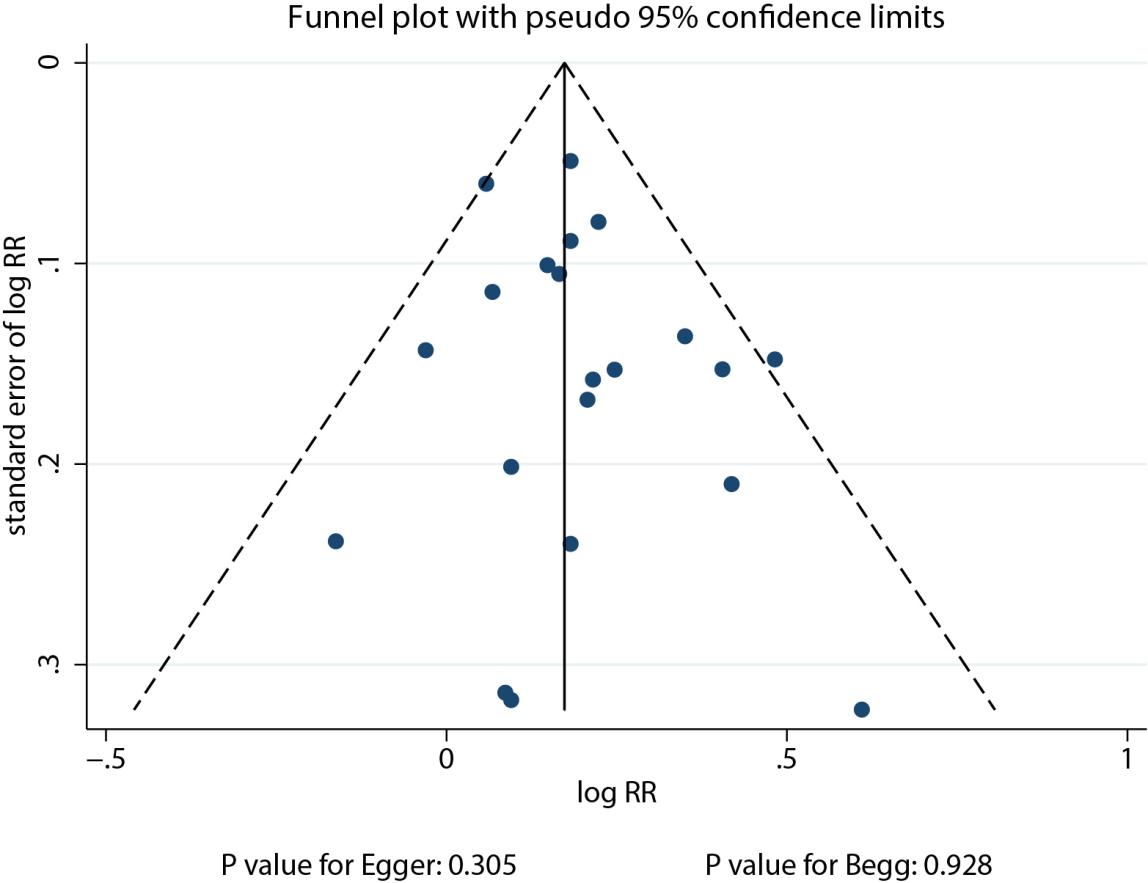


Figure S8. Funnel plot for the relation between proceed meat intake and CRC risk


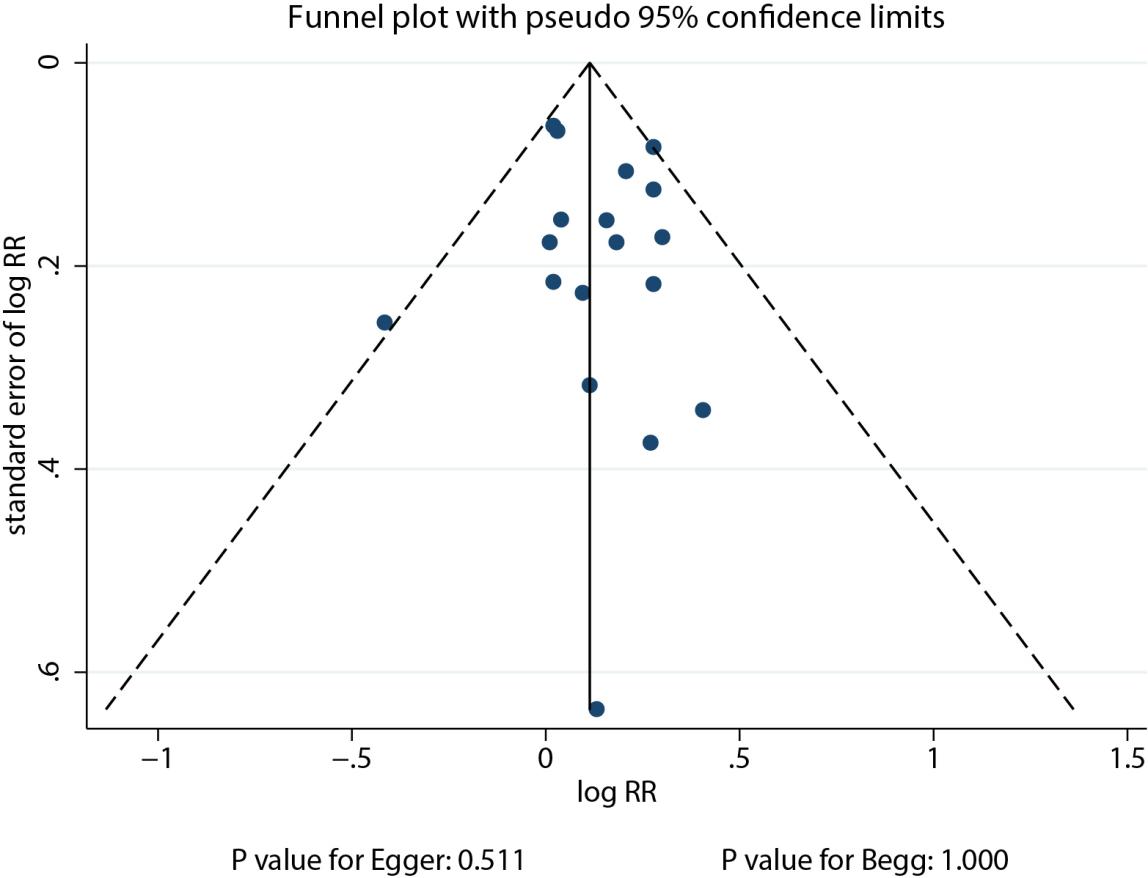


Figure S9. Funnel plot for the relation between total red and processed meat intake and CRC risk


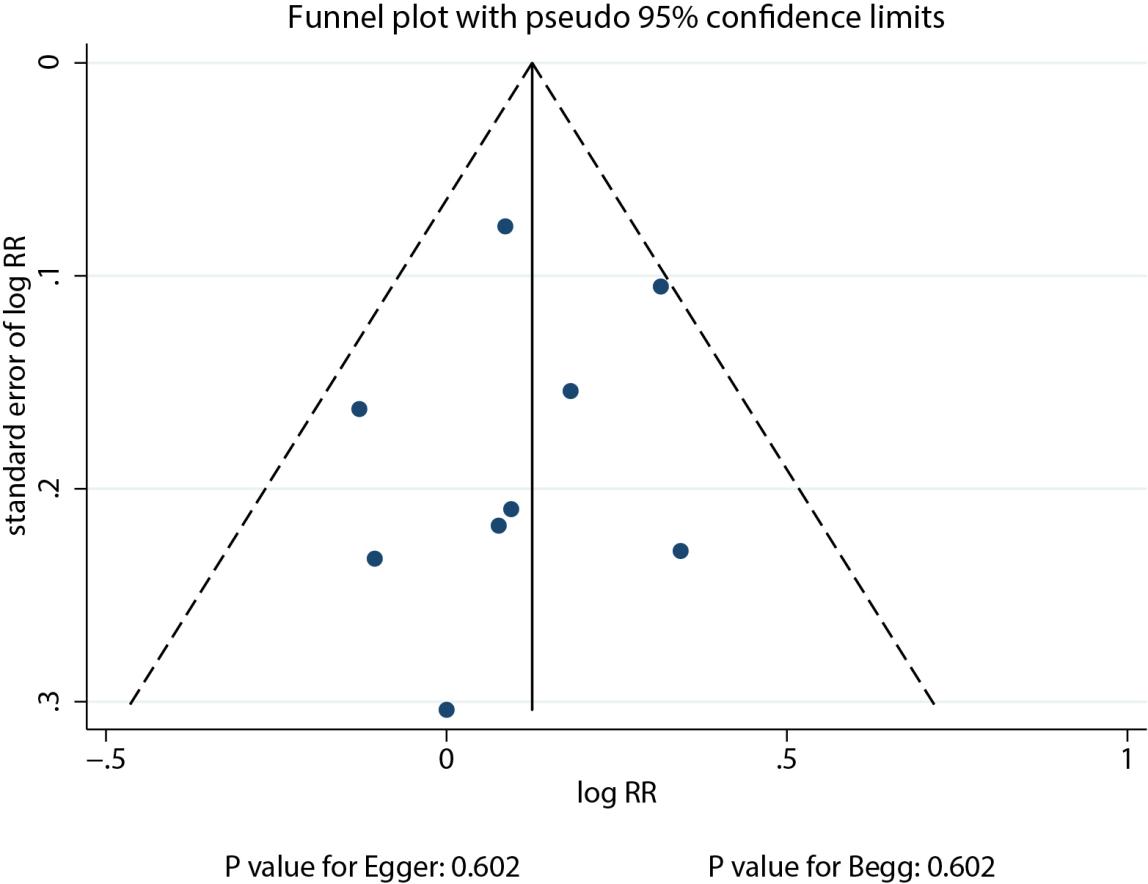


Figure S10. Funnel plot for the relation between red meat intake and CC risk


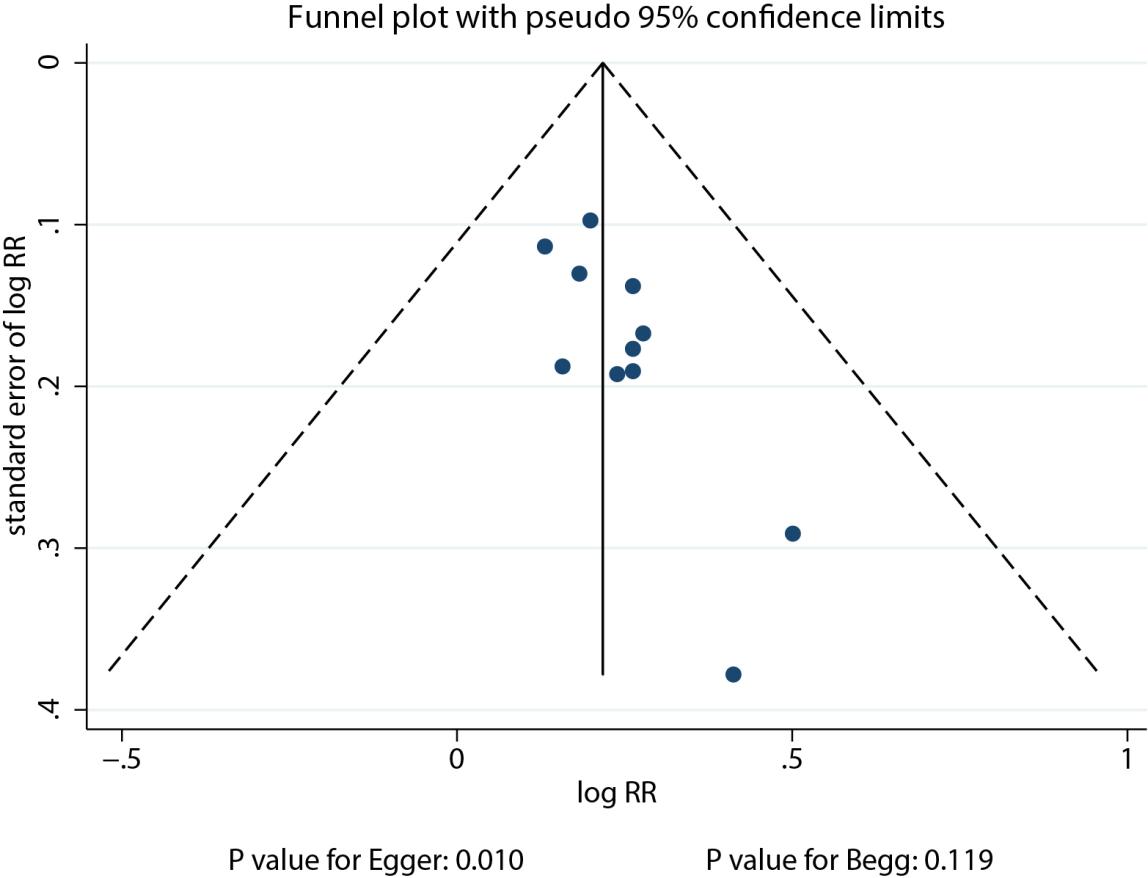


Figure S11. Funnel plot for the relation between proceed meat intake and CC risk


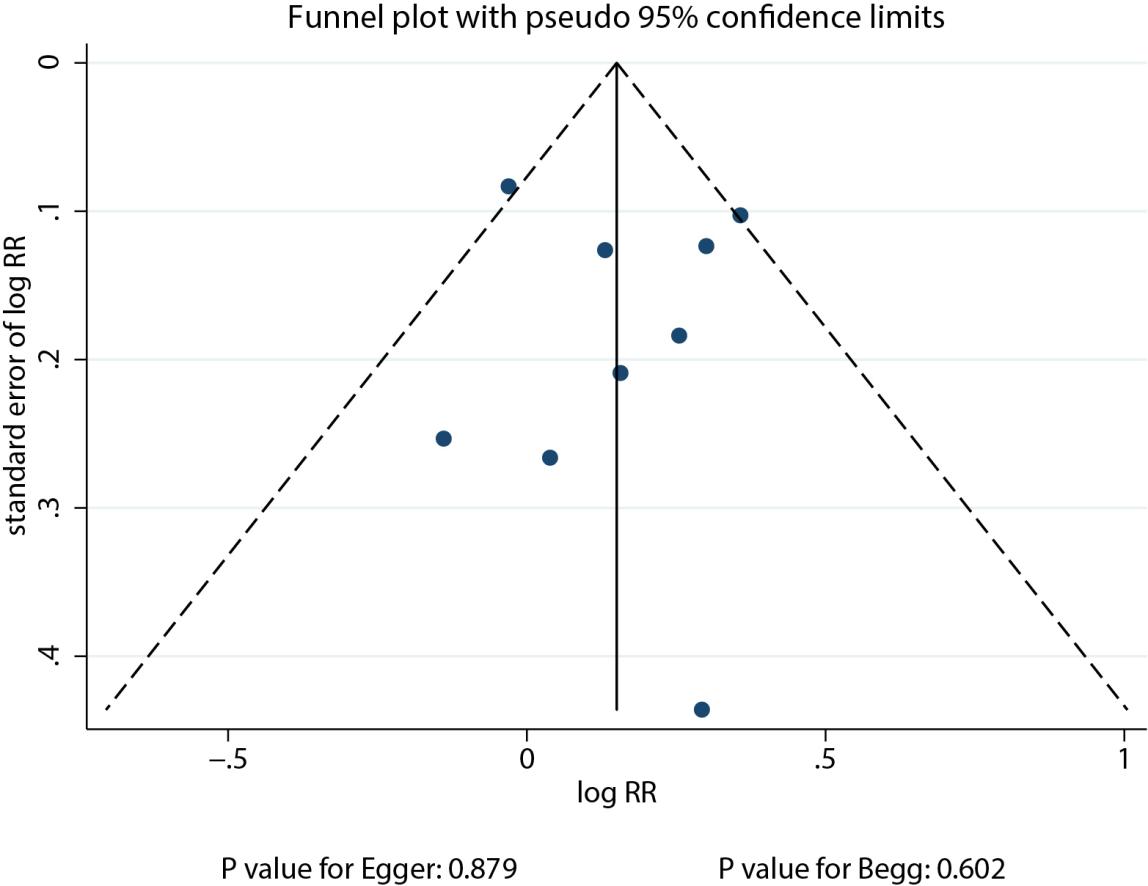


Figure S12. Funnel plot for the relation between total red and processed meat intake and CC risk


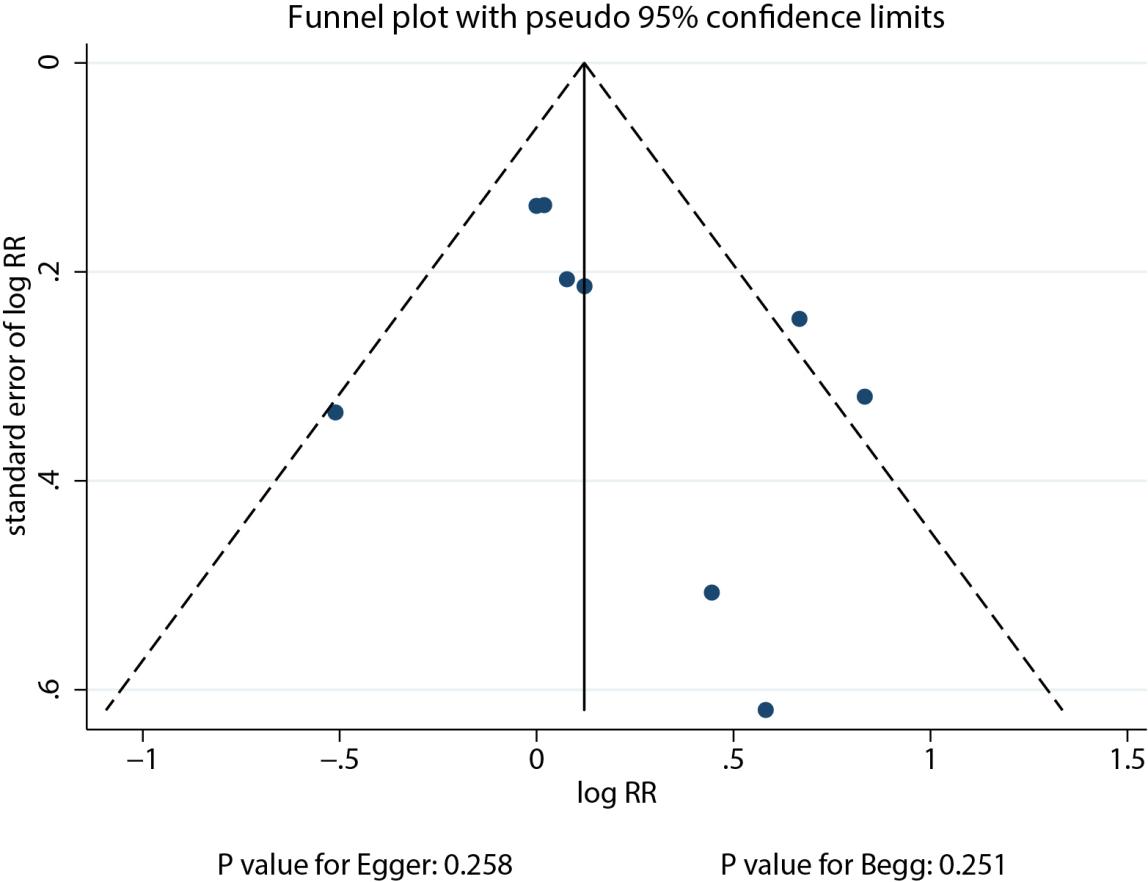


Figure S13. Funnel plot for the relation between red meat intake and RC risk


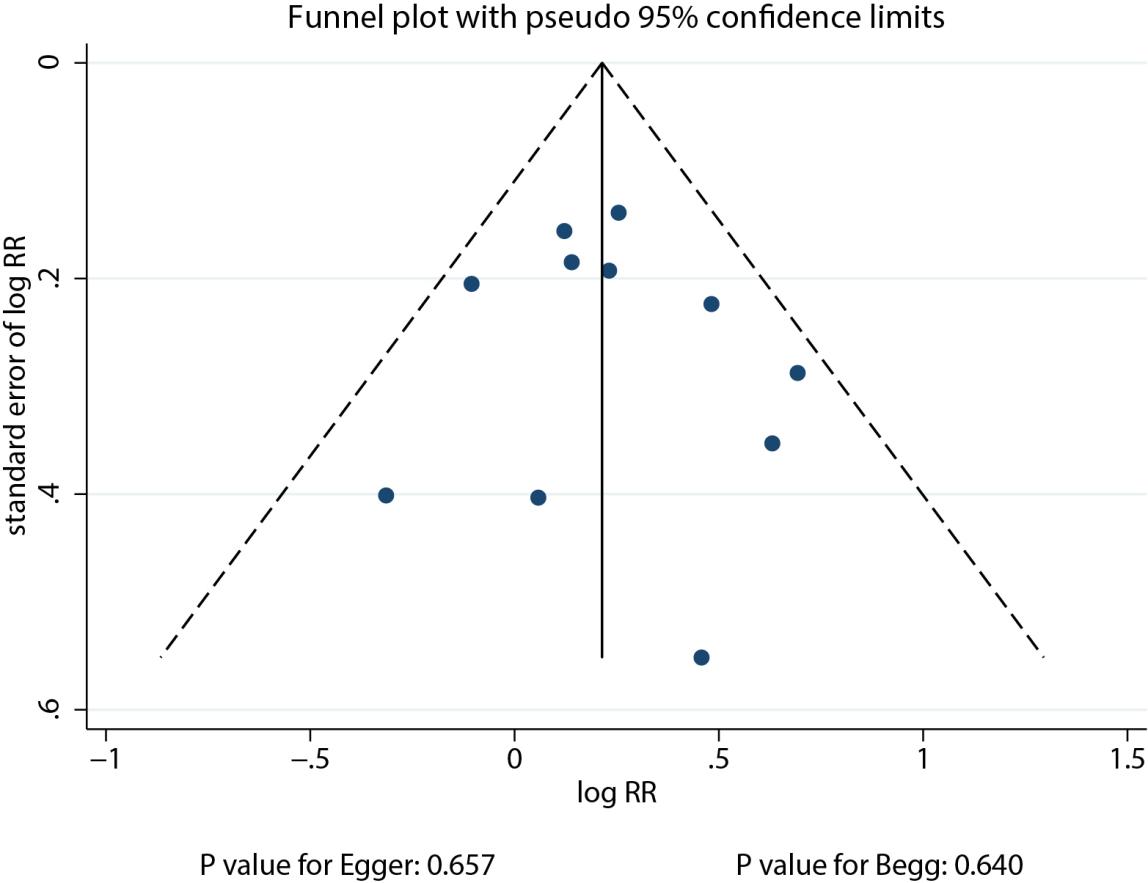


Figure S14. Funnel plot for the relation between proceed meat intake and RC risk


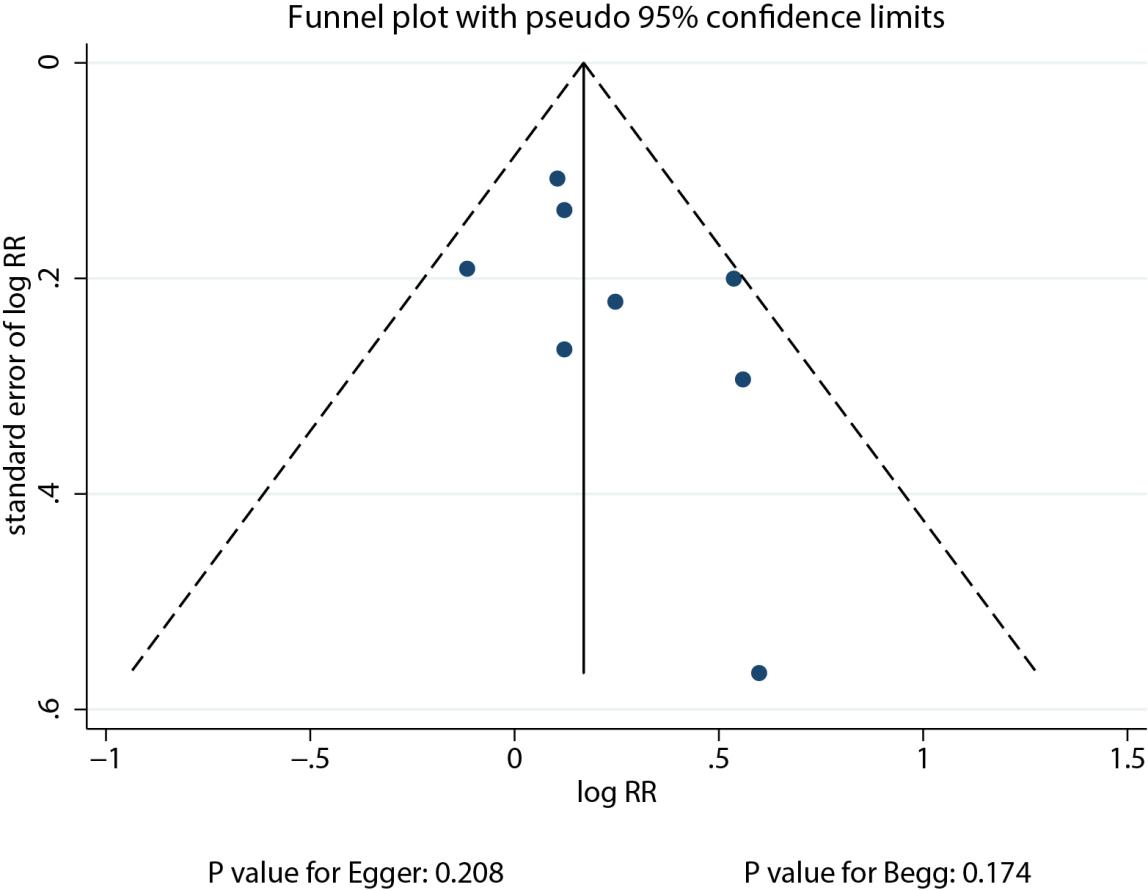


Figure S15. Funnel plot for the relation between total red and processed meat intake and RC risk


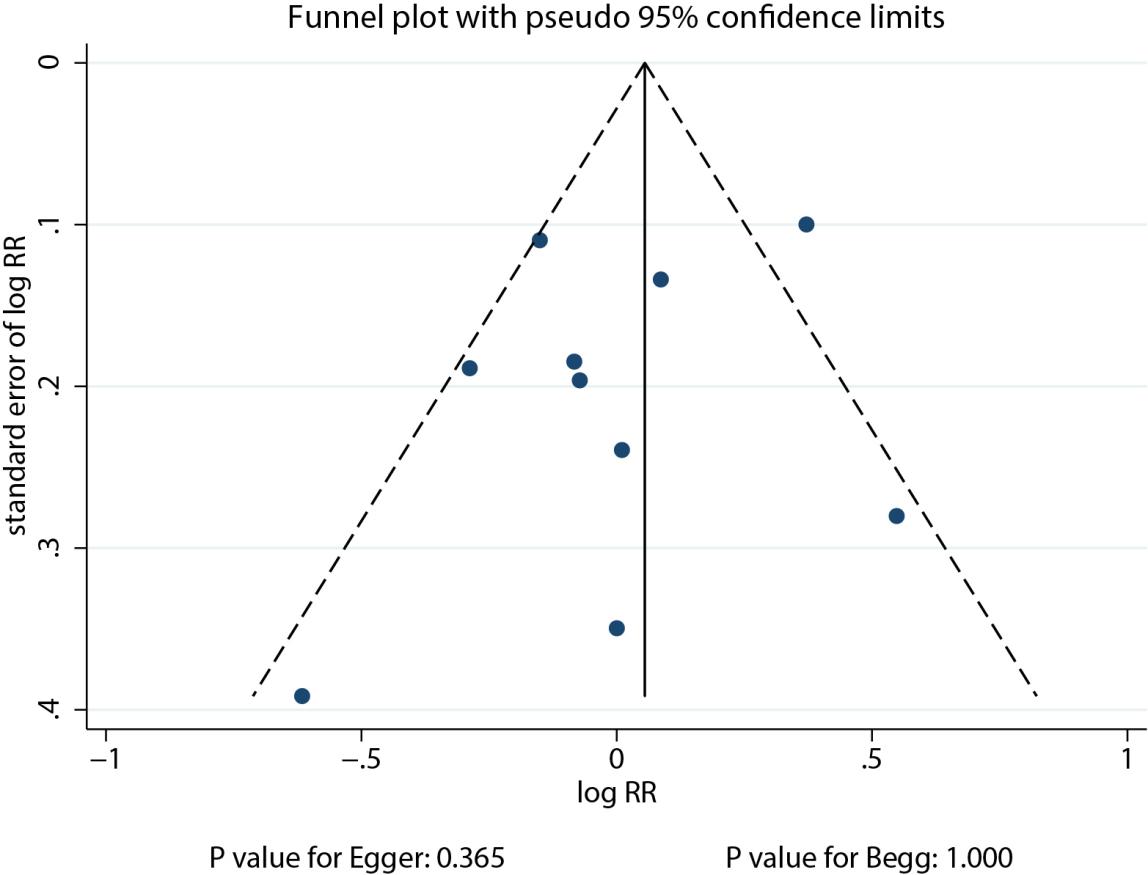


Figure S16. Funnel plot for the relation between red meat intake and PC risk


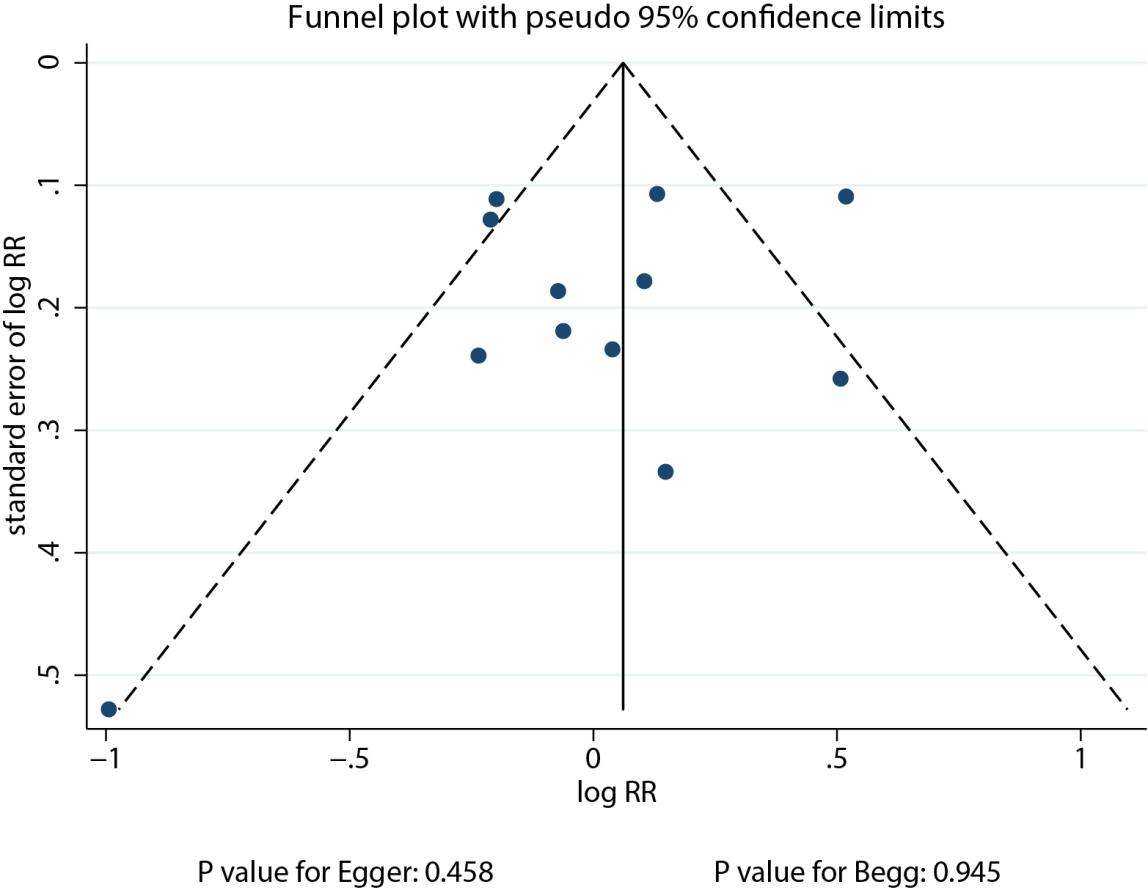


Figure S17. Funnel plot for the relation between proceed meat intake and PC risk


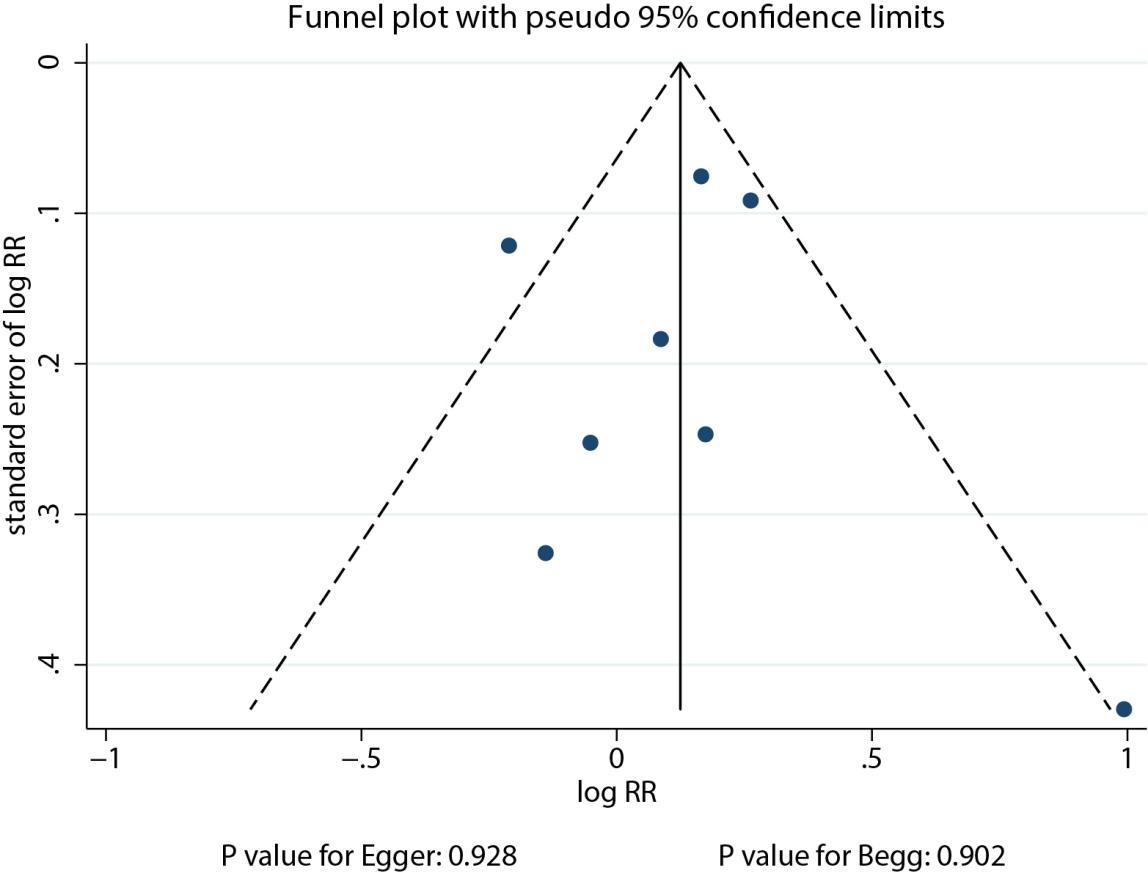


Figure S18. Funnel plot for the relation between total red and processed meat intake and PC risk


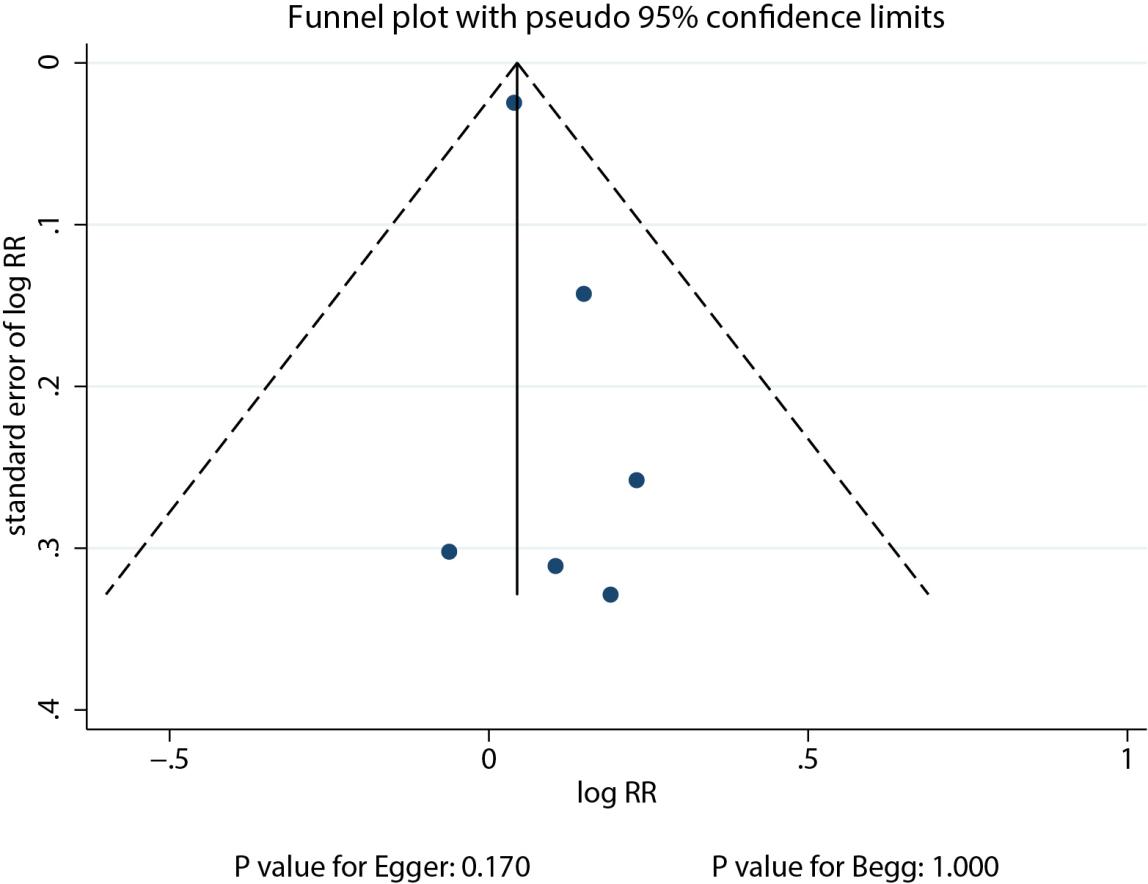


Figure S19. Funnel plot for the relation between red meat intake and HCC risk


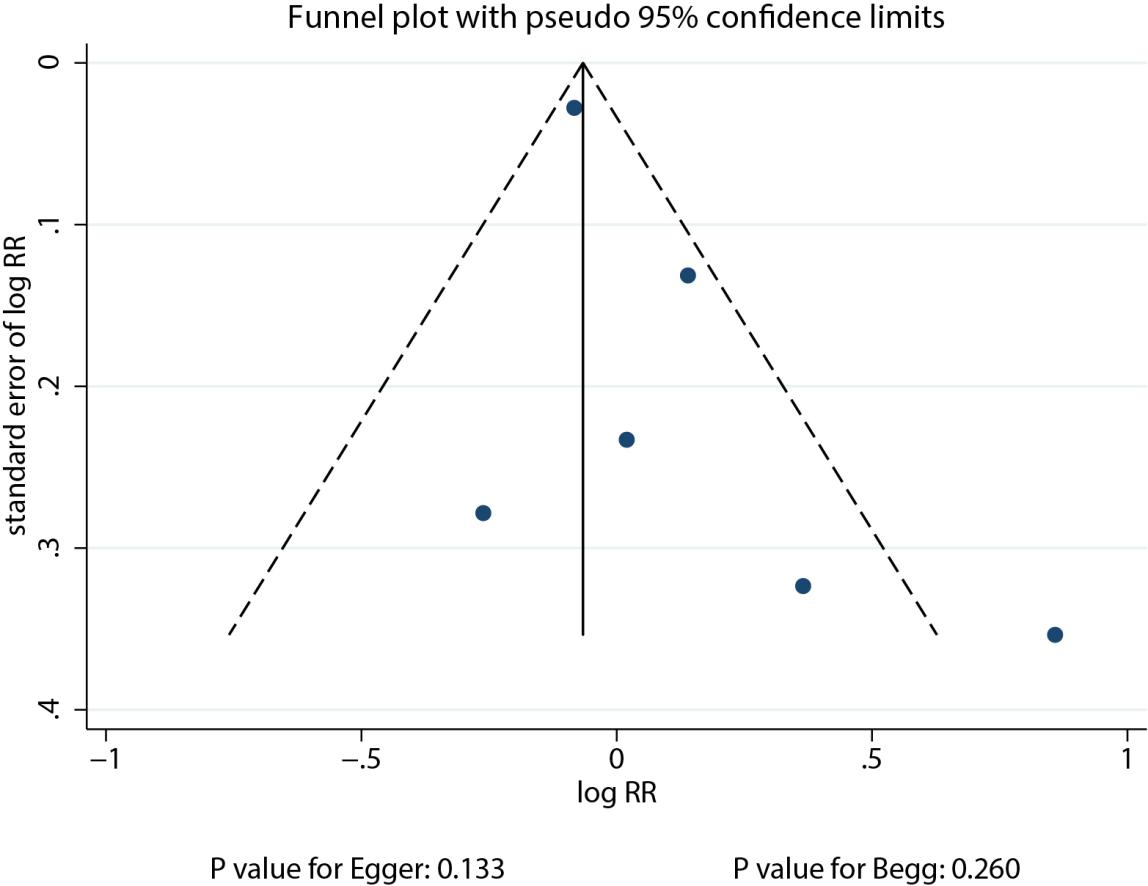


Figure S20. Funnel plot for the relation between proceed meat intake and HCC risk


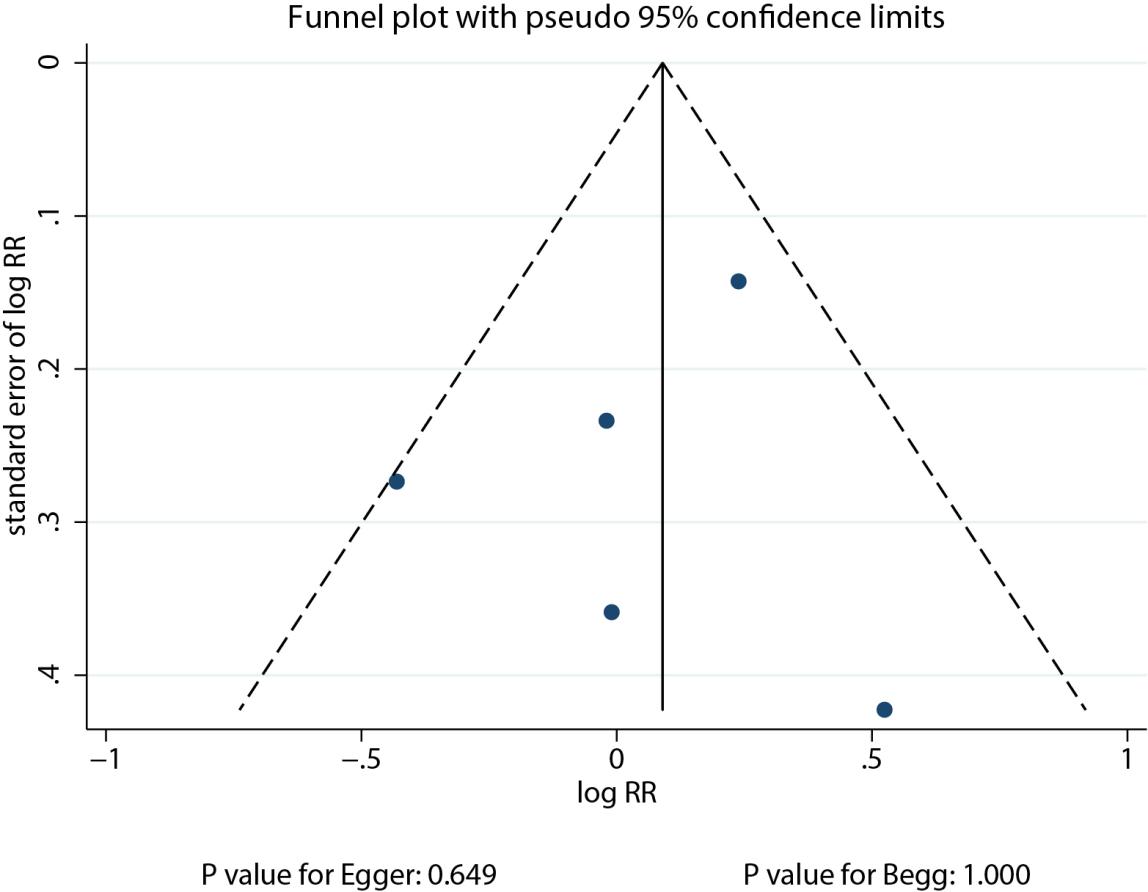


Figure S21. Funnel plot for the relation between total red and processed meat intake and HCC risk
